# Antioxidant and Antitumor Activities of New Synthesized Aromatic *C*-Nucleoside Derivatives

**DOI:** 10.3390/molecules19045163

**Published:** 2014-04-22

**Authors:** Mohamed M. El Sadek, Nagwa S. Abd El-Dayem, Seham Y. Hassan, Mohamed A. Mostafa, Galila A. Yacout

**Affiliations:** 1 Chemistry Department, Faculty of Science, Alexandria University, Alexandria 21231, Egypt; E-Mails: nagwa_abdeldayem@yahoo.com (N.S.A.E.-D.); sehamyassen@yahoo.com (S.Y.H.); dr_abdel_zaher@hotmail.com (M.A.M.); 2 Biochemistry Department, Faculty of Science, Alexandria University, Alexandria 21231, Egypt; E-Mail: galila_69@yahoo.com

**Keywords:** oxoindoline, isopropylidene, pyrrole, indole, isoindole, oxadiazole, pyrazole, antioxidant

## Abstract

The carbohydrazide **1** was used as the precursor for the synthesis of a number of new aromatic *C*-nucleosides containing 1,3,4-oxadiazole **7**, [1,3,4]oxadiazolo[2,3-a]isoindole **10b** and pyrazole units **18**. On the other hand, the thiosemicarbazone **20** was used as the key intermediate for synthesis of 1,3,4-oxadiazole and 1,2,4-triazole-3-thione derivatives **21** and **23**. The antioxidant activities of the prepared compounds were evaluated. The carbohydrazide **1** in particular was found to have potent antioxidant and antitumor activity.

## 1. Introduction

A number of nucleoside analogues have been found to show a broad spectrum of biological effects such as antifungal [1,2], antibacterial [1,2,3], antitumor [3,4,5], antiviral [3,4,6,7,8,9,10,11,12,13,14] anti-inflammatory [15] and analgesic [15] activities. Moreover, 2'-deoxy-2'-fluoro-2'-*C*-methyl nucleoside analogues have showed promising activity against HCV replication [16]. In addition, nucleoside derivatives display inhibition of glucose-6-phosphatase and showed antihyperglycemic effects [17], as well as inhibition of SAH hydrolase [18]. *C*-Nucleosides are a subtype of these compounds that are of great interest owing to their potential biological activity together with their higher stabilities than that of the corresponding *N*-nucleosides. In light of these interesting biological activities and continuation of our research work to explore potent bioactive nitrogen containing molecules [1,2], some aromatic *C*-nucleoside derivatives were prepared and characterized by analytical and spectral methods.

## 2. Results and Discussion

### 2.1. Chemistry

**Scheme 1:**
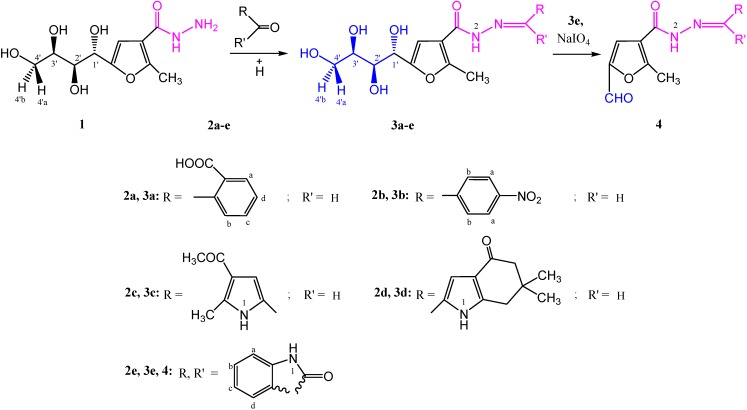
Synthesis of carbohydrazones **3a**–**e** and **4**.

Condensation of the carbohydrazide derivative **1B** [19] with carbonyl compounds **2a**–**e**, afforded the corresponding carbohydrazone derivatives **3a**–**e** in 88%–100% yields. Their structures were confirmed by IR, ^1^H-NMR, two dimentional ^1^H-NMR (COSY), ^13^C-NMR and mass spectral data. The ^1^H-NMR spectrum (DMSO-*d*_6_) of compound **3c**, for example, showed five singlets around δ 11.94, 11.14, 8.13, 6.83 and 6.74, supporting the presence of NH_(1)_, NH_(2)_ (D_2_O exchangeable), azomethine (CH=N), CH_(pyrrole)_ and CH_(furan)_ protons, respectively. Signals of the sugar protons of these derivatives **3a**–**e** were assigned from the 2D ^1^H-NMR spectrum of compound **3c**, and the characteristic chemical shifts as compared with those reported for carbohydrazones [1], whereby, four doublets appearing at δ 5.14, 4.75, 4.60, 4.46 ppm were assigned to 1'-OH, H-1', 2'-OH and 3'-OH, respectively, and a triplet at δ (4.35) ppm for 4'-OH. Two multiplets at δ 3.56–3.50 and 3.42–3.40 ppm were assigned for H-2' overlapped with H-3' and H-4'a, and the other multiplet for H-4'b. A broad singlet at δ 2.47 ppm corresponded to the acetyl protons (COCH_3_), followed by two singlets at δ 2.43 and 2.28 ppm due to methyl protons at the position-2 of the furan ring and CH_3__(pyrrole)_, respectively (see Experimental Section and Scheme 1).

Periodate oxidation of **3e** afforded the corresponding 5-formyl derivative **4**, whose infrared spectrum showed the aldehyde carbonyl functional group at γ 1,689 cm^−1^. In addition the ^1^H-NMR spectrum (DMSO-*d*_6_) of this product, showed a high field singlet at δ 9.60 ppm for the aldehyde proton (CHO). The mass spectrum showed the molecular ion peak at *m/z* 297 (M^+^, 22.54%).

**Scheme 2:**
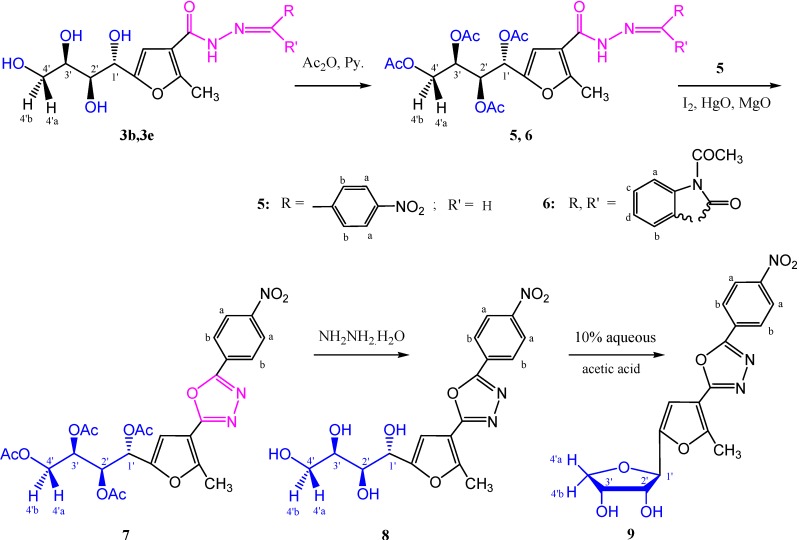
Synthesis of aromatic *C*-nucleosides **5**–**9**.

Acetylation of compounds **3b** and **3e**, afforded the corresponding acetyl derivatives **5** and **6**, in 95% and 100% yield, respectively, as indicated by their spectral data. Oxidative cycization [1,2,20] of the carbohydrazone **5**, gave the 1,3,4-oxadiazole derivative **7** which lacked the carbonyl and NH bands in its infrared spectrum. The mass spectrum gave the parent ion peak at *m/z* 559 (M^+^, 18.83%). (see Experimental Section and Scheme 2).

On the other hand, boiling of the tetrayltetraacetate **7** with hydrazine hydrate resulted in the corresponding tetrahydroxybutyl derivative **8** in 97% yield. Its IR spectrum showed the hydroxyl groups (OH) at γ 3387–3200 cm^−1^, while the corresponding ^1^H-NMR spectrum revealed the four hydroxyl protons at δ 5.23–4.37 ppm, and the mass spectrum showed the molecular ion peak in agreement with expected molecular weight of compound **8**. Moreover, dehydrative cyclization of the tetraol derivative **8** with aqueous acetic acid (10%) afforded the aromatic *C*-nucleoside **9** in 63% yield, as detected from its spectral data. Its ^1^H-NMR spectrum (DMSO-*d*_6_) showed only two D_2_O-exchangeable hydroxyl protons for 2'-OH and 3'-OH, as two doublets at δ 5.16 and 5.03 ppm, respectively. The mass spectrum showed the expected molecular ion peak in agreement with its structure.

**Scheme 3:**
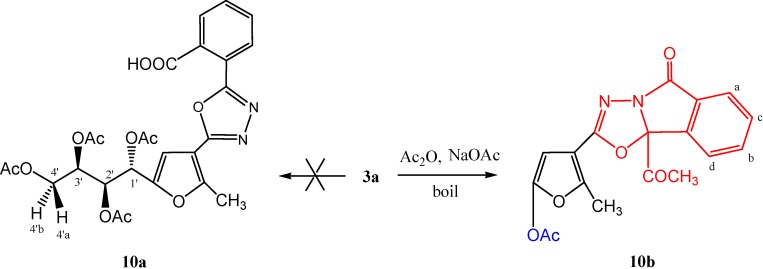
Synthesis of [1,3,4]oxadiazolo[2,3-a]-isoindole **10b**.

**Scheme 4:**
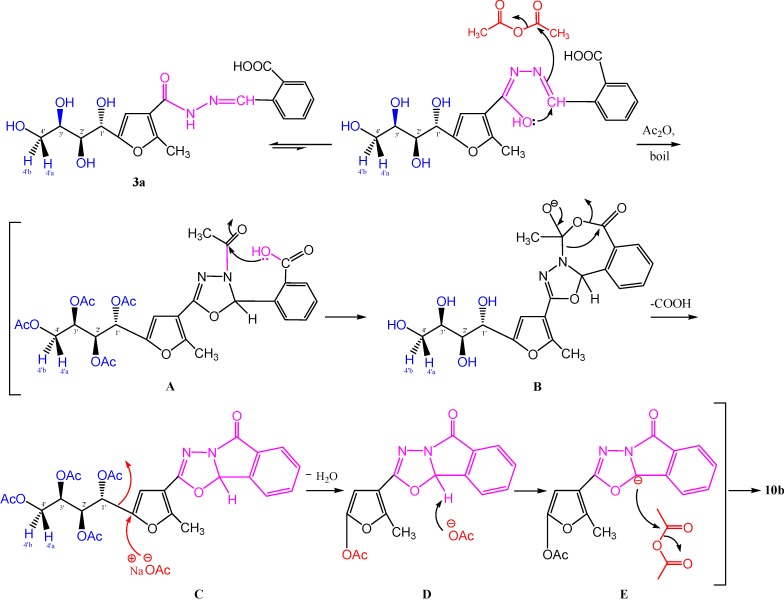
Proposed mechanism for formation of [1,3,4]oxadiazolo[2,3-a]isoindole **10b**.

Condensative cyclization of carbohydrazone **3a** with acetic anhydride in the presence of anhydrous sodium acetate gave a cyclized product that according to physical and chemical studies, could not be reconciled with the structure of **10a**, that but rather was compatible with that of [1,3,4]oxadiazolo-[2,3-a]isoindole **10b**. The infrared spectrum of this compound showed the disappearance of the carboxylic acid hydroxyl group, sugar hydroxyl groups, and CONH absorption bands. It showed instead an acetoxyl (OAc) group at γ 1,744 cm^−1^ and carbonyl groups at γ 1,725 and 1,712 cm^−1^. Its ^1^H-NMR spectrum (CDCl_3_) showed the disappearance of signals corresponding to the sugar protons at the δ 3.00–5.00 ppm region, and only displayed the aromatic protons as a doublet at δ 7.84 (*J* = 7.65 Hz) for Ar-H_(a)_, a triplet at δ 7.65 (*J* = 7.65 Hz) for Ar-H_(b)_, a triplet at δ 7.56 (*J* = 7.65 Hz) for Ar-H_(c)_, and a doublet at δ 7.53 (*J* = 7.65 Hz) for Ar-H_(d)_, followed by a singlet attributed to CH_(furan)_ at δ 7.00 ppm. Three singlet signals that appeared in the upper field region at δ 2.56, 2.27 and 2.14 ppm were attributed to the CH_3__(furan)_, COCH_3_ and *O*-acetyl protons, respectively. It is noteworthy that the integration of the OAc protons (δ 2.14 ppm), indicated only one *O*-acetyl group, in accord with structure **10b**. Moreover, the proposed mechanism for formation of **10b** may proceed as illustrated in Scheme 3 and Scheme 4.

In addition, condensation of anhydro derivative **11** [2] with *p*-nitrobenzaldehyde, indoline-2,3-dione (isatin) and d-galactose in acidic medium afforded the corresponding aromatic *C*-nucleosides **12**–**14**, respectively. Compounds **12** and **13** were also obtained by acid-catalyzed dehydrative cyclization of **3b** and **3e**, respectively. Their structures were deduced from the respective spectral data. The signals of the sugar protons of anhydro structures **12** and **13** were assigned from the characteristic chemical shifts as compared with those reported for diol derivatives [2]. Although the coupling constant value (*J*_1',2'_ = 6.85 Hz) of **12** cannot define the anomeric configuration [21], however, it could be β- in accordance with the configuration its precursor [2]. On the other hand, the anomeric configuration of **13** can be ascertained from the large observed coupling constant value (*J*_1',2'_ = 9.00 Hz) which indicates a *trans* arrangement of the base moiety and the 2'-hydroxyl group, *i.e.*, β-d-configuration.

Furthermore, acetylation of **12** and **13**, afforded the acetylated structures **15** and **16**, in 79% and 67% yields, respectively. The infrared spectra showed OAc absorption bands at γ 1,753, 1,745 cm^−1^, respectively. The ^1^H-NMR spectra (CDCl_3_) of these products revealed two singlet signals at δ 2.00–2.11 ppm attributed to two *O*-acetyl groups. Their mass spectra showed the expected molecular ion peaks in agreement with their proposed structures.

**Scheme 5:**
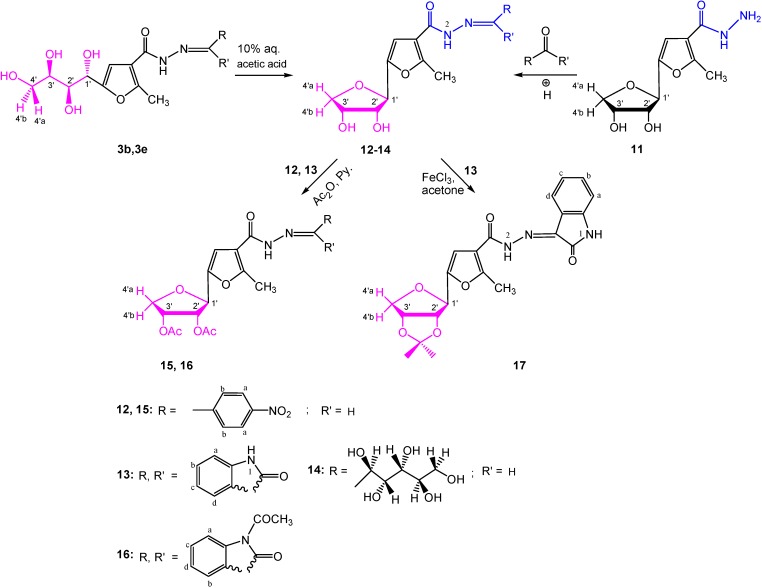
Synthesis of aromatic *C*-nucleosides **12**–**17**.

The isopropylidene derivative **17** has been prepared from **13** in yield 88%. Its anomeric configuration was confirmed from the zero coupling constant value (*J*_1',2'_ = 0.00 Hz), as a β-d-configuration [2,21,22]. The mass spectrum showed the expected molecular ion peak in agreement with its structure (Scheme 5).

**Scheme 6:**
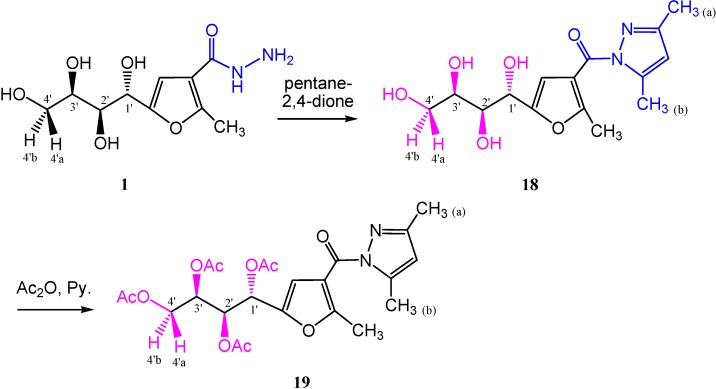
Synthesis of pyrazole derivatives **18** and **19**.

The pyrazole derivative **18** was obtained in 100% yield from the reaction of carbohydrazide **1** with pentane-2,4-dione as previously reported on other systems [23]. The infrared spectrum showed the disappearance of absorption bands corresponding to NH and NH_2_. Its ^1^H-NMR spectrum (DMSO-*d*_6_), revealed three singlets at δ 6.18, 2.48 and 2.16 ppm for CH_(pyrazole)_, CH_3(pyrazole-a)_ and CH_3(pyrazole-b)_ protons, respectively. The molecular ion peak recorded in the mass spectrum was in accordance with its molecular weight. Furthermore, the *O*-acetyl derivative **19** was prepared, in which the signals of the sugar protons of this product were assigned from its 2D ^1^H-NMR spectrum (Scheme 6).

**Scheme 7:**
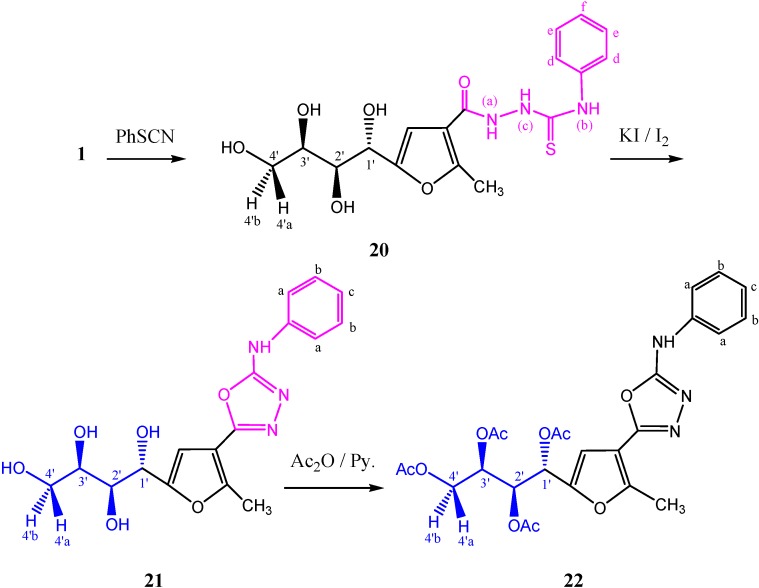
Synthesis of 1,3,4-oxadiazoles **21** and **22**.

Moreover, condensation of **1** with phenyl isothiocyanate gave the corresponding thiosemicarbazide derivative **20** [24]. Intramolecular cyclization of this thiosemicarbazide using an improved procedure involving treatment with potassium iodide and iodine in the presence of sodium hydroxide [25] resulted in 1,3,4-oxadiazole product **21** [24] in 95% yield. The tetra-*O*-acetyl derivative **22** was obtained in 85% yield, the signals of the sugar protons of this product were assigned from its 2D ^1^H-NMR spectrum, the mass spectrum showed the molecular ion peak at *m/z* 529 (M^+^, 19.12%), and ^13^C-NMR (CDCl_3_) spectrum confirmed the structure (Scheme 7).

**Scheme 8:**
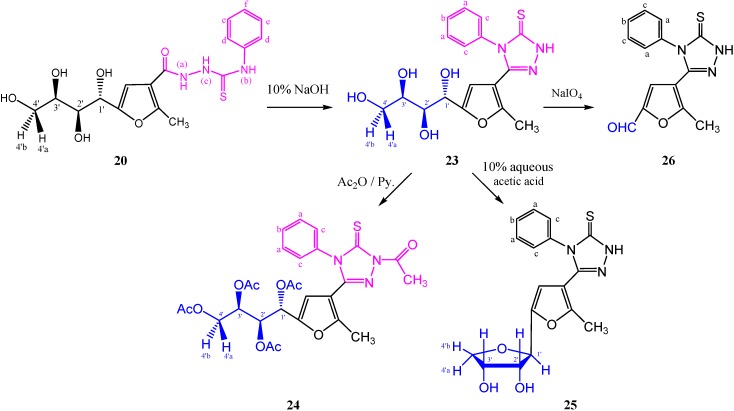
Synthesis of 4-phenyl-2*H*-1,2,4-triazole-3(4*H*)-thiones **23**–**26**.

Alternatively, heating the thiosemicarbazide **20** with aqueous sodium hydroxide (10%) [25] gave a product **23**, whose infrared spectrum showed a C=N absorption at γ 1,624 cm^−1^ with the disappearance of the CONH absorption. Moreover, acetylation of **23**, gave 5-(5-(1',2',3',4'-tetraacetoxybutyl)-2-methylfuran-3-yl)-4-phenyl-2*-N*-acetyl-1,2,4-triazole-3(4*H*)-thione (**24**) in 97% yield. The ^1^H-NMR spectrum (CDCl_3_) revealed the disappearance of the NH proton and showed a singlet due to *N*-acetyl protons at δ 2.77 ppm, followed by three singlets at δ 2.01, 1.99, and 1.97 ppm for four *O*-acetyl groups. The mass spectrum showed a molecular ion peak in accordance with its molecular formula (Scheme 8).

Dehydration of **23**, afforded 2',3'-diol **25** in yield of 80% (Scheme 8). The anomeric configuration of **25** can be confirmed as β-d-configuration (*J*_1',2'_ = 9.00 Hz). The mass spectrum showed the expected molecular ion peak at *m/z* 359 (M^+^, 20.21%). The characteristic alcohol M-H_2_O peak appeared at *m/z* 341 (20.21), while the M-SH peak was seen at *m/z* 326 (20.21). The loss of a furanose moiety (C_4_H_8_O_3_) from the molecular ion appeared at *m/z* 255 (20.91). Furthermore, periodate oxidation of **23**, afforded the corresponding 2-carbaldehyde derivative **26**.

### 2.2. Bioactivity Screening of New Synthesized Aromatic C-Nucleosides

#### 2.2.1. Antioxdant Activity Screening (Using the DPPH Assay)

**Figure 1:**
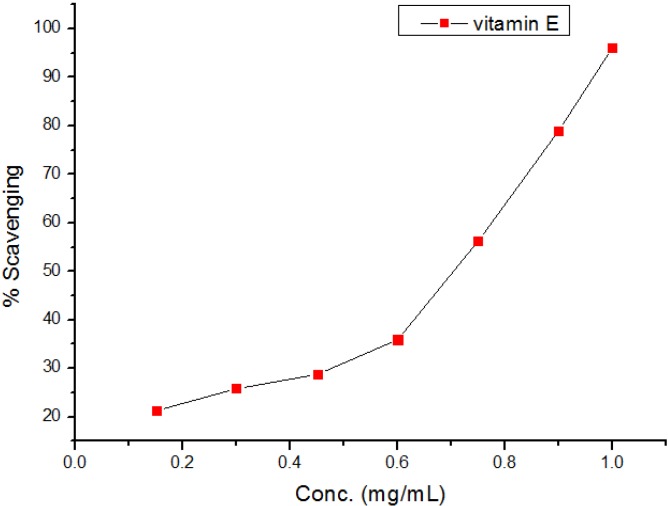
Free radical scavenging activity of vitamin E.

**Figure 2:**
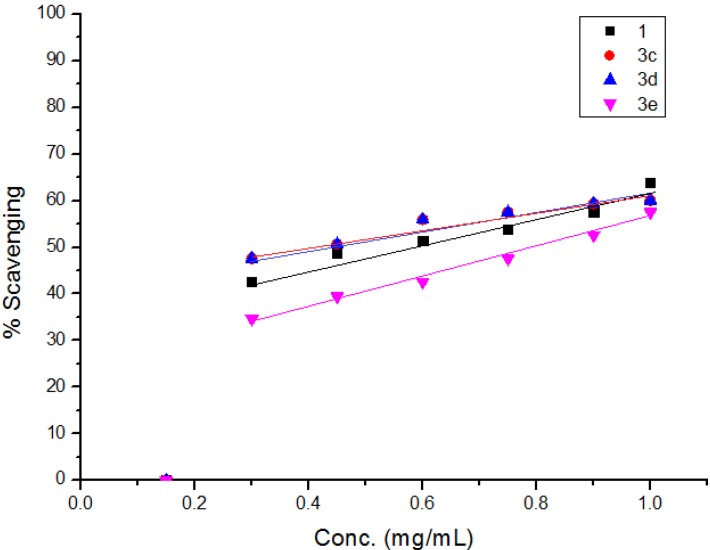
Free radical scavenging activity of compounds **1**, **3c**–**e**.

**Figure 3:**
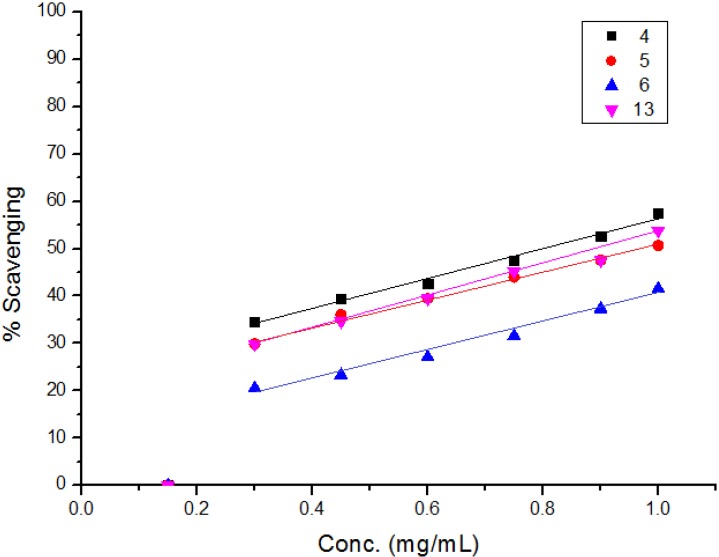
Free radical scavenging activity of compounds **4**–**6**, **13**.

**Figure 4:**
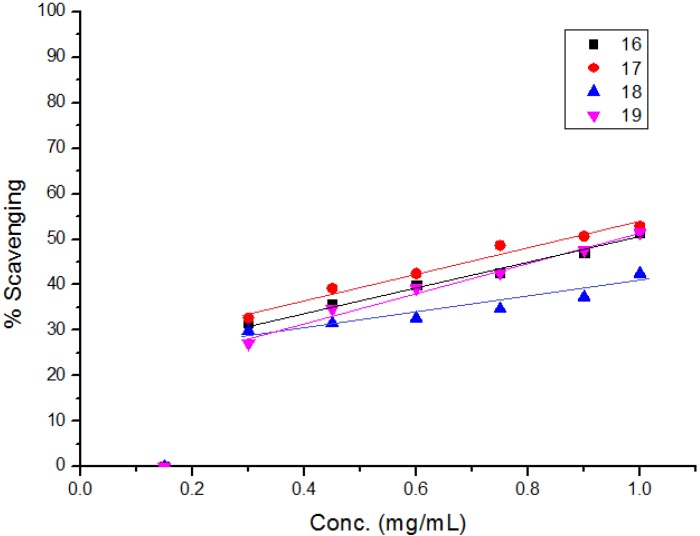
Free radical scavenging activity of compounds **16**–**19**.

**Figure 5:**
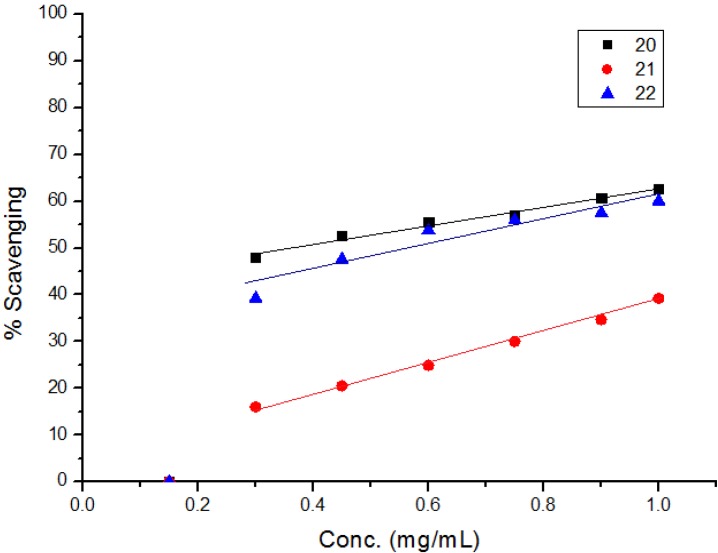
Free radical scavenging activity of compounds **20**–**22**.

**Figure 6:**
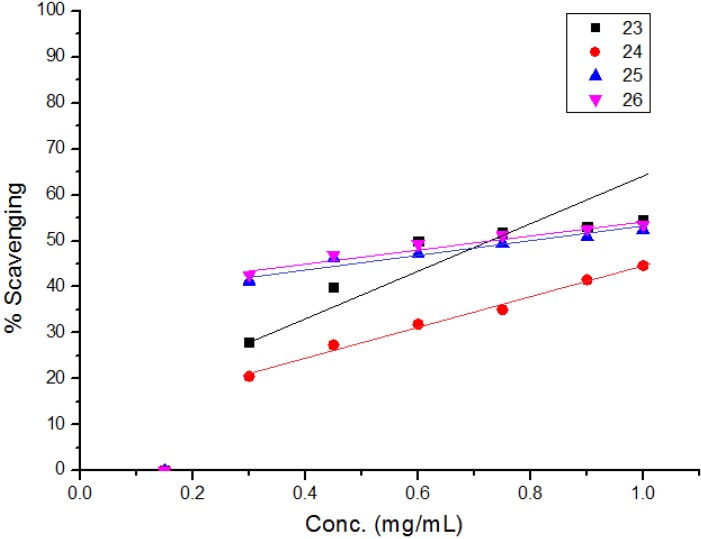
Free radical scavenging activity of compounds **23**–**26**.

The diphenylpicrylhydrazyl (DPPH) assay method is based on the reduction of the free radical DPPH with an odd electron which gives a maximum absorption at 517 nm. When antioxidants react with DPPH, giving DPPD-H the absorbance decreases due to decolorization with respect to the number of electrons captured. EC_50_ values for each examined compound as well as standard preparations were calculated according to the method Shahwar *et al.* [26]. A lower EC_50_ value is associated with a higher radical scavenging activity. As shown in Table 1 and Table 2 and Figure 1, Figure 2, Figure 3, Figure 4, Figure 5 and Figure 6 the DPPH radical scavenging activities of the prepared compounds **1**, **3c**–**e**, **4**–**6**, **13**, and **16**–**26** in terms of EC_50_ values were the highest in the case of compounds **20**, **3c**, **3d**, **1** and **22** (0.380, 0.418, 0.448, 0.590 and 0.590 mg, respectively) compared to the EC_50_ of vitamin E used as standard (0.705). Meanwhile nearly the same activities were revealed in case of compounds **23** and **26** (0.720 and 0.725 mg), respectively. In addition, moderate activities were shown for compounds **3e**, **4**, **17** and **25** (0.800, 0.800, 0.825 and 0.815 mg), respectively. Lower activities were observed in case of compounds **5**, **6**, **13**, **16**, **18**, **19**, **21** and **24** with EC_50 values_ equal to 0.960, ˃ 1.000, 0.900, 0.980, ˃ 1.000, 0.930, ˃ 1.000, ˃ 1.000 mg, respectively, compared to the standard, see Table 2.

**Table 1:** Absorbance and free radical scavenging activities of tested compounds.

Conc.(mg/ mL)	Compound 1		Compound 3c		Compound 3d		Compound 3e	
	Absorbance	% Scavenging	Absorbance	% Scavenging	Absorbance	% Scavenging	Absorbance	% Scavenging
0.150	0.000	0.00	0.000	0.00	0.000	0.00	0.000	0.00
0.300	0.369	42.61	0.420	47.58	0.337	47.58	0.420	34.68
0.450	0.330	48.67	0.399	50.69	0.317	50.69	0.399	39.50
0.600	0.312	51.47	0.380	55.98	0.283	55.98	0.380	42.61
0.750	0.297	53.81	0.369	57.54	0.273	57.54	0.369	47.58
0.900	0.273	57.54	0.330	59.40	0.261	59.40	0.330	52.56
1.000	0.232	63.91	0.297	60.03	0.257	60.03	0.297	57.54
**Conc. (mg/ mL)**	**Compound 4**		**Compound 5**		**Compound 6**		**Compound 13**	
	**Absorbance**	**% Scavenging**	**Absorbance**	**% Scavenging**	**Absorbance**	**% Scavenging**	**Absorbance**	**% Scavenging**
0.150	0.000	0.00	0.000	0.00	0.000	0.00	0.000	0.00
0.300	0.420	34.68	0.451	29.86	0.511	20.52	0.451	29.86
0.450	0.389	39.50	0.411	36.08	0.493	23.32	0.420	34.68
0.600	0.369	42.61	0.389	39.50	0.469	27.06	0.389	39.50
0.750	0.337	47.58	0.360	44.01	0.440	31.57	0.352	45.25
0.900	0.305	52.56	0.337	47.58	0.403	37.32	0.337	47.58
1.000	0.273	57.54	0.317	50.69	0.376	41.52	0.297	53.81
**Conc. (mg/ mL)**	**Compound 16**		**Compound 17**		**Compound 18**		**Compound 19**	
	**Absorbance**	**% Scavenging**	**Absorbance**	**% Scavenging**	**Absorbance**	**% Scavenging**	**% Scavenging**	**Absorbance**
0.150	0.000	0.00	0.000	0.00	0.000	0.00	0.000	0.00
0.300	0.440	31.57	0.433	32.65	0.451	29.86	0.469	27.06
0.450	0.413	35.76	0.391	39.19	0.440	31.57	0.420	34.68
0.600	0.387	39.81	0.370	42.45	0.433	32.65	0.391	39.19
0.750	0.369	42.61	0.330	48.67	0.420	34.68	0.369	42.61
0.900	0.341	46.96	0.317	50.69	0.403	37.32	0.337	47.58
1.000	0.312	51.47	0.303	52.87	0.370	42.45	0.312	51.47
**Conc. (mg/ mL)**	**Vitamin E**		**Compound 20**		**Compound 21**		**Compound 22**	
	**Absorbance**	**% Scavenging**	**Absorbance**	**% Scavenging**	**Absorbance**	**% Scavenging**	**% Scavenging**	**Absorbance**
0.150	0.756	21.25	0.000	0.00	0.000	0.00	0.000	0.00
0.300	0.712	25.83	0.335	47.90	0.540	16.01	0.391	39.19
0.450	0.684	28.75	0.305	52.56	0.511	20.52	0.337	47.58
0.600	0.615	35.93	0.285	55.67	0.483	24.88	0.297	53.81
0.750	0.420	56.25	0.276	57.07	0.450	30.01	0.283	55.98
0.900	0.202	78.95	0.252	60.80	0.420	34.68	0.273	57.54
1.000	0.037	96.14	0.240	62.67	0.391	39.19	0.257	60.03
**Conc. (mg/ mL)**	**Compound 23**		**Compound 24**		**Compound 25**		**Compound 26**	
	**absorbance**	**% scavenging**	**absorbance**	**% scavenging**	**absorbance**	**% scavenging**	**absorbance**	**% scavenging**
0.150	0.000	0.00	0.000	0.00	0.000	0.00	0.000	0.00
0.300	0.463	27.99	0.511	20.52	0.378	41.21	0.369	42.61
0.450	0.387	39.81	0.467	27.37	0.346	46.18	0.341	46.96
0.600	0.322	49.92	0.438	31.88	0.340	47.12	0.326	49.30
0.750	0.310	51.78	0.418	34.99	0.325	49.45	0.314	51.16
0.900	0.302	53.03	0.376	41.52	0.316	50.85	0.305	52.56
1.000	0.292	54.58	0.356	44.63	0.306	52.41	0.299	53.49

**Table 2:** EC_50_ values of the prepared compounds **1**, **3c**–**3e**, **4**–**6**, **13**, **16**–**26**.

Cpd no.	EC_50_ (mg)	Cpd no.	EC_50_ (mg)
**Vitamin E**	0.705	**17**	0.825
**1** [1]	0.590	**18**	˃1.000
**3c**	0.418	**19**	0.930
**3d**	0.448	**20**	0.380
**3e**	0.800	**21**	˃1.000
**4**	0.800	**22**	0.590
**5**	0.960	**23**	0.720
**6**	˃1.000	**24**	˃1.000
**13**	0.900	**25**	0.815
**16**	0.980	**26**	0.725

The obtained data revealed a potential antioxidant activity of all examined compounds but with different EC_50_ values compared to the standard, especially compound **20** which has a distinct thiourea group. In addition, compounds **3c** and **3d** revealed higher antioxidant activities as compared to the standard due to the acidic protons in the pyrrole and indole, respectively, that can be easily oxidized.

#### 2.2.2. Anticancer Activity Screening (Cytotoxicity Against Three Cancer Cell Lines)

**Figure 7:**
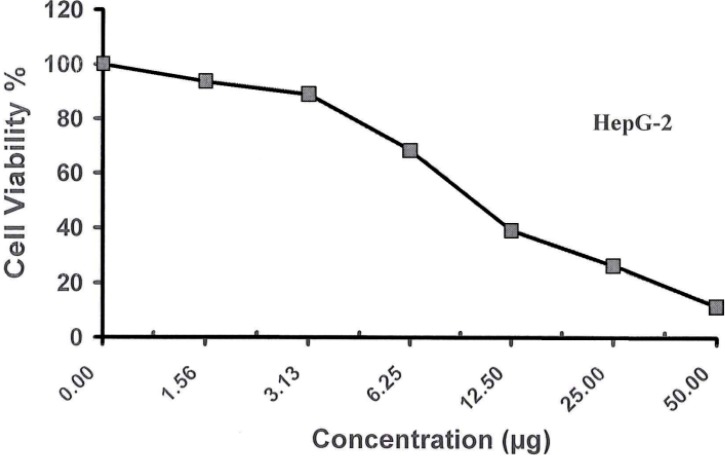
Viability activity against HepG-2 of compound **1**.

**Figure 8:**
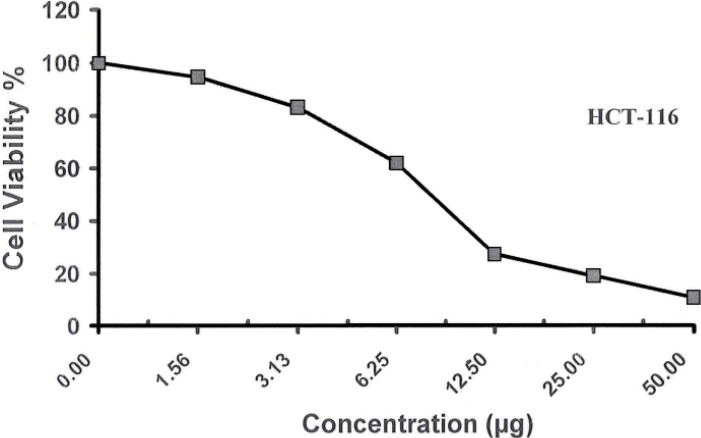
Viability activity against HCT-116 of compound **1**.

**Figure 9:**
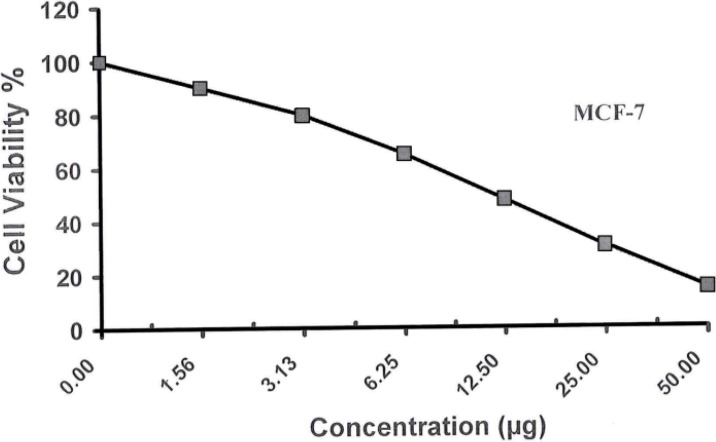
Viability activity against MCF-7 of compound **1**.

Different concentrations (50–1.56 µg/mL) of the examined compound **1** were used to screen their cytotoxicity against Human Breast Adrenocarcinoma Cells (MCF-7), Human Colon Carcinoma Cells (HCT) and Human Hepatocellular Liver Carcinoma Cells (HepG-2). Cytotoxic effects of these compounds on the cell viability of the cancer cell lines were observed, as shown in Table 3 and Table 4 and Figure 7, Figure 8 and Figure 9. The obtained data revealed that the carbohydrazide **1** has excellent cell growth inhibitory effects on HepG-2, HCT and MCF-7 with IC_50_s equal to 10.200, 8.400 and 11.700 µg, respectively compared to the IC_50_ of the doxorubicin (1.200, 0.469) and vinblastine (6.100) standards used, see Table 5.

**Table 3:** Effect of standard compounds on cell viability using cytotoxic assay.

Conc. (μg/mL)	Doxorubicin for HCT	Doxorubicin for HepG-2	Vinblastine for MCF-7
	Viability %	Viability %	Viability %
50.000	6.82	10.95	7.82
25.000	8.89	14.29	15.18
12.500	14.83	16.90	29.6
6.250	16.16	21.03	48.75
3.125	22.28	30.32	60.35
1.560	34.64	48.25	76.24
0.780	45.78	57.44	….
0.390	51.08	….	….
0.000	100.00	100.00	100.00

**Table 4:** Effect of different concentrations of compound **1** on cell viability using cytotoxic assay.

Conc. (μg/mL)	Viability % for HCT	Viability % for HepG-2	Viability % for MCF-7
50.000	10.68	11.56	14.68
25.000	19.09	26.34	30.49
12.500	27.25	39.18	47.84
6.250	61.87	68.47	64.98
3.125	83.08	89.05	79.82
1.560	94.62	93.78	90.18
0.000	100.00	100.00	100.00

**Table 5:** IC_50_ of compound **1** on cell viability using cytotoxic assay compared to standards.

Standard	IC_50_ (μg/mL)		
	HCT	HepG-2	MCF-7
	0.469	1.20	6.10
Cpd. 1	8.400	10.20	11.70

## 3. Experimental

### 3.1.General Procedures

Melting points were determined with a Melt-temperature apparatus and are uncorrected. TLC was performed on Baker-Flex silica gel 1B-F plates and the spots were detected by UV light absorption. IR spectra were recorded on a Perkin Elmer spectrometer. ^1^H-NMR and ^13^C-NMR were recorded on JEOL JNM ECA 500 MHz and 300 MHz instruments using tetramethylsilane as an internal standard. Mass spectra were recorded on a GCMS DI Analysis Shimadzu Qp-2010 Plus. Solutions were evaporated under diminished pressure unless otherwise stated. ChemDraw-Ultra-8.0 has been used in generating the names of the prepared compounds.

### 3.2. Chemistry

#### 3.2.1. 5-(1',2',3',4'-Tetrahydroxybutyl)-2-methylfuran-3-carbohydrazide (**1**)

Mp 200–201 °C (Lit. [1], 198 °C); IR(KBr) cm^−1^: 3400–3137 (OH, NH), 1674 (C=O); ^1^H-NMR (DMSO-*d*_6_); δ: 2.46 (s, 3H, CH_3(furan)_), 3.34–3.40 (m, 3H, H-4'b, NH_2_ with H_2_O of DMSO), 3.44–3.58 (m, 3H, H-3', H-2', H-4'a), 4.36 (t, 1H, 4'-OH, *J*_4',OH_ = 5.35 Hz, D_2_O-exchangeable), 4.48 (d, 1H, 3'-OH, *J*_3',OH_ = 6.85 Hz, D_2_O-exchangeable), 4.61 (d, 1H, 2'-OH, *J*_2',OH_ = 5.35 Hz, D_2_O-exchangeable), 4.73 (d, 1H, H-1', *J*_1',2'_ = 5.35 Hz), 5.14 (d, 1H, 1'-OH, *J*_1',OH_ = 6.10 Hz, D_2_O-exchangeable), 6.70 (s, 1H, CH_(furan)_), 9.79 (s, 1H, NH, D_2_O-exchangeable).

#### 3.2.2. General Method for the Synthesis of Carbohydrazones **3a**–**e**

A mixture of 3-carbohydrazide **1** (1.923 mmoL) and carbonyl compound (1.923 mmoL) was heated under reflux in ethanol (10 mL) containing a few drops of acetic acid for 1 h. The products **3a**–**e** that separated were filtered off and dried.

*2-((5-(1',2',3',4'-Tetrahydroxybutyl)-2-methylfuran-3-carboylimino)methyl)benzoic acid* (**3a**). Obtained in 91% yield from carbohydrazide **1** (1.923 mmoL) and 2-formylbenzoic acid (1.923 mmoL); recrystallized from ethanol as white crystals; mp 209–210 °C; Rf: 0.85 (chloroform–methanol; 5:1; *v/v*); IR (KBr) cm^−1^: 3429–3305 (OH), 3187 (NH), 1703 (CO-acid), 1653 (CO-amide), 1593 (C=N); ^1^H-NMR (DMSO-*d*_6_); δ: 2.50 (s, 3H, CH_3(furan)_), 3.46–3.49 (m, 1H, H-4'b), 3.53–3.59 (m, 3H, H-3', H-2', H-4'a), 4.36 (bs, 1H, 4'-OH, D_2_O-exchangeable), 4.49 (bs, 1H, 3'-OH, D_2_O-exchangeable), 4.62 (bs, 1H, 2'-OH, D_2_O-exchangeable), 4.76 (s, 1H, H-1'), 5.16 (bs, 1H, 1'-OH, *J*_1',OH_ = 6.00 Hz, D_2_O-exchangeable), 6.83 (s, 1H, CH_(furan)_), 7.47 (t, 1H, Ar-H_(d)_, *J* = 7.65 Hz), 7.60 (t, 1H, Ar-H_(c)_, *J* = 7.65 Hz), 7.85 (d, 1H, Ar-H_(b)_, *J* = 7.65 Hz), 8.01 (d, 1H, Ar-H_(a)_, *J* = 7.65 Hz), 9.08 (s, 1H, CH=N), 11.60 (s, 1H, NH, D_2_O-exchangeable), 12.58 (bs, 1H, COOH, D_2_O-exchangeable); MS: *m/z* (%), 393 (25.33, M^+^+1), 392 (28.00, M^+^), 357 (25.78), 347 (23.11), 343 (40.00), 330 (32.89), 300 (40.89), 262 (46.67), 251 (52.44), 220 (36.44), 208 (37.33), 183 (46.67), 177 (25.33), 154 (32.44), 151 (32.44), 146 (38.67), 137 (32.89), 102 (33.78), 91 (38.22), 89 (100.00), 77 (43.11), 65 (38.22), 50 (28.00); Anal. Calcd for C_18_H_2_0N_2_O_8_: C, 55.10; H, 5.14; N, 7.14%; found: C, 55.20; H, 5.00; N, 7.23%.

*N-(4-Nitrobenzylidene)-5-(1',2',3',4'-tetrahydroxybutyl)-2-methylfuran-3-carbohydrazide* (**3b**). Obtained in 98% yield from carbohydrazide **1** (1.923 mmoL) and *p*-nitrobenzaldehyde (1.923 mmoL); recrystallized from ethanol as yellow crystals. Rf: 0.38 (chloroform–methanol, 5:1, *v/v*); mp 163–164 °C; IR (KBr): 3447–3202 (OH, NH), 1664 (C=O), 1585 (C=N); ^1^H-NMR (DMSO-*d*_6_); δ: 2.51 (s, 3H, CH_3(furan)_), 3.38–3.42 (m, 1H, H-4'b), 3.46–3.51 (m, 1H, H-4'a ), 3.52–3.54 (m, 1H, H-3'), 3.55–3.60 (m, 1H, H-2'), 4.36 (t, 1H, 4'-OH *J*_4',OH_ = 5.35 Hz, D_2_O exchangeable), 4.49 (d, 1H, 3'-OH, *J*_3',OH_ = 6.90 Hz, D_2_O exchangeable), 4.61 (d, 1H, 2'-OH, *J*_2',OH_ = 5.35 Hz, D_2_O exchangeable), 4.77 (d, 1H, H-1',*J*_1',2'_ = 6.10 Hz), 5.18 (d, 1H, 1'-OH, *J*_1',OH_ = 6.90 Hz, D_2_O exchangeable), 6.80 (s, 1H, CH_(furan)_), 7.92 (d, 2H, Ar-H_(b)_, *J* = 7.65 Hz), 8.26 (d, 2H, Ar-H_(a)_, *J* = 8.45 Hz), 8.46 (s, 1H, CH=N), 11.67 (s, 1H, NH, D_2_O exchangeable); MS: *m/z* (%), 394 (20.12, M^+^+1), 393 (26.33, M^+^), 375 (16.86), 355 (28.11), 347 (24.85), 307 (28.99), 306 (25.44), 287 (25.74), 271 (16.27), 245 (15.98), 227 (23.08), 221 (28.99), 211 (47.04), 151 (100.00), 143 (20.71), 138 (21.60), 137 (26.63), 123 (52.66), 113 (24.26), 95 (42.31), 94 (42.31), 81 (26.33), 77 (24.26), 76 (19.53), 65 (22.49), 58 (23.96), 53 (37.28); Anal. Calcd for C_17_H_19_N_3_O_8_: C, 51.91; H, 4.87; N, 10.68%; found: C, 51.95; H, 4.81; N, 10.55%.

*N-((4-Acetyl-5-methyl-1H-pyrrol-2-yl)methylene)-5-(1',2',3',4'-tetrahydroxybutyl)-2-methylfuran-3-carbohydrazide* (**3c**). Obtained in 88% yield from carbohydrazide **1** (1.923 mmoL) and 4-acetyl-5-methyl-1*H*-pyrrole-2-carbaldehyde (1.923 mmoL); recrystallized from ethanol as white crystals; Rf: 0.52 (chloroform–methanol, 5:1, *v/v*); mp 160–161 °C; IR (KBr): 3409 (OH), 3262 (NH-pyrrole), 3222 (NH-amide), 1642 (2C=O), 1619 (C=N); ^1^H-NMR (DMSO-*d*_6_); δ: 2.28 (s, 3H, CH_3(pyrrole)_), 2.43 (s, 3H, CH_3(furan)_), 2.47 (bs, 3H, COCH_3_ with DMSO), 3.42–3.40 (m, 1H, H-4'b), 3.56–3.50 (m, 3H, H-3', H-2', H-4'a), 4.35 (t, 1H, 4'-OH, *J*_4'-OH_ = 6.00 Hz, D_2_O-exchangeable), 4.46 (d, 1H, 3'-OH, *J*_3',OH_ = 6.00 Hz, D_2_O-exchangeable), 4.60 (d, 1H, 2'-OH, *J*_2',OH_ = 6.00 Hz, D_2_O-exchangeable), 4.75 (d, 1H, H-1', *J*_1',2'_ = 6.00 Hz), 5.14 (d, 1H, 1'-OH, *J*_1',OH_ = 6.00 Hz, D_2_O-exchangeable), 6.74 (s, 1H, CH_(furan)_), 6.83 (s, 1H, CH_(pyrrole)_), 8.13 (s, 1H, CH=N), 11.14 (s, 1H, NH_(2)_, D_2_O exchangeable), 11.94 (s, 1H, NH_(1)_, D_2_O exchangeable); ^13^C-NMR (DMSO-*d*_6_); δ: 13.88 (CH_3-pyrrole_ and CH_3-furan_), 28.80 (COCH_3_), 63.84, 66.56, 71.54 and 73.25 for (C-4', C-3', C-2' and C-1'), 105.90, 115.35, 115.80, 122.17, 125.93, 139, 139.5, 155.10 and 155.89 for (pyrrole and furan carbons and CH=N), 159.86 (CO-NH), 193.86 (COCH_3_); MS: *m/z* (%), 395 (53.39, M^+^+2), 394 (69.49, M^+^+1), 393 (44.07, M^+^), 361 (57.63), 359 (71.19), 358 (55.93), 357 (59.32), 344 (62.71), 340 (55.93), 323 (64.41), 311 (60.17), 259 (59.32), 254 (57.63), 242 (79.66), 233 (53.39), 228 (72.88), 223 (66.95), 206 (55.08), 195 (71.19), 191 (64.41), 185 (60.17), 180 (64.41), 158 (59.32), 148 (66.10), 123 (66.95), 116 (64.41), 108 (55.93), 106 (68.64), 91 (100.00), 75 (55.93), 63 (60.17); Anal. Calcd for C_18_H_23_N_3_O_7_: C, 54.96; H, 5.89; N, 10.68%; found: C, 54.91; H, 5.77; N, 10.56%.

*N-((4,5,6,7-Tetrahydro-6,6-dimethyl-4-oxo-1H-indol-2-yl)methylene)-5-(1',2',3',4'-tetrahydroxybutyl)-2-methylfuran-3-carbohydrazide* (**3d**). Obtained in 90% yield from carbohydrazide **1** (1.923 mmoL) and 4,5,6,7-tetrahydro-6,6-dimethyl-4-oxo-1*H*-indole-2-carbaldehyde (1.923 mmoL); recrystallized from ethanol as white crystals; Rf: 0.7 (chloroform–methanol, 4:1, *v/v*); mp 194–195 °C; IR (KBr): 3345 (OH), 3257 (NH- indole), 3158 (NH-amide), 1656 (2C=O), 1618 (C=N); ^1^H-NMR (DMSO-*d*_6_); δ: 0.99 (s, 6H, 2CH_3(indole)_), 2.20 (s, 2H, CH_2(indole)_), 2.47 (s, 3H, CH_3(furan)_ with DMSO), 2.67 (s, 2H, CH_2(indole)_), 3.38–3.43 (m, 1H, H-4'b), 3.49–3.56 (m, 3H, H-3', H-2', H-4'a), 4.31 (t, 1H, 4'-OH, *J*_4',OH_ = 6.00 Hz, D_2_O-exchangeable), 4.43 (d, 1H, 3'-OH, *J*_3',OH_ = 6.00 Hz, D_2_O-exchangeable), 4.56 (d, 1H, 2'-OH, *J*_2',OH_ = 6.00 Hz, D_2_O-exchangeable), 4.75 (d, 1H, H-1', *J*_1',2'_ = 6.00 Hz), 5.10 (d, 1H, 1'-OH, *J*_1',OH_ = 9.00 Hz, D_2_O-exchangeable), 6.60 (s, 1H, CH_(furan)_), 6.74 (s, 1H, CH_(indole)_), 8.17 (s, 1H, CH=N), 11.15 (s, 1H, NH_(2)_, D_2_O exchangeable), 11.90 (s, 1H, NH_(1)_, D_2_O exchangeable); MS: *m/z* (%), 434 (19.10, M^+^+1), 433 (73.03, M^+^), 432 (73.03), 378 (69.66), 359 (88.76), 348 (76.40), 343 (83.15), 329 (70.79), 301 (70.79), 296 (73.03), 278 (73.03), 276 (70.79), 275 (79.78), 274 (73.03), 255 (94.38), 250 (69.66), 244 (91.01), 215 (70.79), 214 (76.40), 205 (69.66), 199 (74.16), 195 (79.78), 191 (83.15), 189 (69.66), 185 (73.03), 180 (70.79), 175 (83.15), 174 (70.79), 165 (79.78), 152 (67.42), 144 (78.65), 122 (70.79), 116 (70.79), 112 (70.79), 89 (78.65), 75 (61.80), 65 (80.90), 52 (100.00); Anal. Calcd for C_21_H_27_N_3_O_7_: C, 58.19; H, 6.28; N, 9.69%; found: C, 58.14; H, 6.10; N, 9.70%.

*5-(1',2',3',4'-Tetrahydroxybutyl)-2-methyl-N-(2-oxoindolin-3-ylidene)furan-3-carbohydrazide* (**3e**). Obtained in 100% yield from carbohydrazide **1** (1.923 mmoL) and isatin (1.923 mmoL); recrystallized from ethanol as yellow needles; mp 206–207 °C; Rf: 0.61 (chloroform–methanol; 5:1; *v/v*); IR (KBr) cm^−1^: 3402 (OH), 3250 (2NH), 1675 (2CO), 1620 (C=N); ^1^H-NMR (DMSO-*d*_6_); δ: 2.55 (s, 3H, CH_3(furan)_), 3.39–3.42 (m, 1H, H-4'b), 3.51–56 (m, 3H, H-3', H-2', H-4'a), 4.32 (t, 1H, 4'-OH, *J*_4',OH_ = 3.00 Hz, D_2_O-exchangeable), 4.53 (d, 1H, 3'-OH, *J*_3',OH_ = 6.00 Hz, D_2_O-exchangeable), 4.58 (d, 1H, 2'-OH, *J*_2',OH_ = 3.00 Hz, D_2_O-exchangeable), 4.79 (d, 1H, H-1', *J*_1',2'_ = 6.00 Hz), 5.20 (d, 1H, 1'-OH, *J*_1',OH_ = 6.00 Hz, D_2_O-exchangeable), 6.52 (s, 1H, CH_(furan)_), 6.93 (d, 1H, Ar-H_(d)_, *J* = 9.00 Hz), 7.07 (t, 1H, Ar-H_(c)_, *J* = 9.00 Hz), 7.35 (t, 1H, Ar-H_(b)_, *J* = 9.00 Hz), 7.55 (d, 1H, Ar-H_(a)_, *J* = 9.00 Hz), 11.25 (bs, 1H, NH_(2)_, D_2_O-exchangeable), 13.40 (bs, 1H, NH_(1)_, D_2_O-exchangeable); MS: *m/z* (%), 391 (11.64, M^+^+2), 390 (13.87, M^+^+1), 389 (11.13, M^+^), 379 (11.99), 374 (12.16), 333 (11.13), 314 (12.55), 302 (12.50), 283 (12.16), 269 (12.67), 268 (12.50), 263 (15.24), 252 (13.53), 236 (13.87), 229 (12.16), 221 (13.53), 196 (13.36), 194 (13.53), 154 (13.36), 149 (16.27), 137 (17.64), 125 (14.73), 124 (18.15), 123 (13.36), 119 (13.87), 115 (14.73), 113 (13.01), 112 (17.12), 111 (23.46), 110 (12.16), 109 (21.58), 107 (13.01), 103 (11.99), 101 (15.75), 100 (12.67), 99 (12.16), 98 (12.67), 97 (38.01), 96 (29.79), 95 (32.19), 94 (15.75), 85 (29.79), 84 (35.79), 83 (44.86), 82 (22.60), 81 (36.64), 79 (20.89), 73 (20.89), 71 (45.03), 70 (32.53), 69 (82.53), 68 (23.97), 67 (38.70), 66 (13.87), 60 (19.52), 57 (100.00), 56 (37.67), 55 (88.01), 54 (24.32); Anal. Calcd for C_18_H_19_N_3_O_7_: C, 55.53; H, 4.92; N, 10.79%; found: C, 55.42; H, 5.00; N, 10.88%.

#### 3.2.3. 5-Formyl-2-methyl-N-(2-oxoindolin-3-ylidene)furan3-carbohydrazide (**4**)

A solution of compound **3e** (3.856 mmol) in distilled water (20 mL) was treated dropwise with a solution of sodium metaperiodate (11.568 mmol) in distilled water (20 mL) under continuous stirring for 3 h, and the formyl derivative that separated out was filtered off, washed with water, and dried. Yield 92%; recrystallized from ethanol as yellow crystals; mp 280 °C; Rf: 0.44 (chloroform–methanol; 30:1; *v/v*); IR (KBr) cm^−1^: 3158 (2NH), 1689 (CHO), 1665 (2CO), 1623 (C=N); ^1^H-NMR (DMSO-*d*_6_); δ: 2.67 (s, 3H, CH_3(furan)_), 6.93 (d, 1H, Ar-H_(d)_, *J* = 9.00 Hz), 7.07 (t, 1H, Ar-H_(c)_, *J* = 9.00 Hz), 7.36 (t, 1H, Ar-H_(b)_, *J* = 9.00 Hz), 7.58 (d, 1H, Ar-H_(a)_, *J* = 9.00 Hz), 7.78 (bs, 1H, CH_(furan)_), 9.60 (s, 1H, CHO), 11.34 (bs, 1H, NH_(2)_, D_2_O exchangeable), 13.48 (bs, 1H, NH_(1)_, D_2_O exchangeable); MS: *m/z* (%), 299 (0.83, M^+^+2), 298 (4.73, M^+^+1), 297 (22.54, M^+^), 269 (7.98), 161 (6.47), 160 (56.39), 159 (11.55), 138 (9.53), 137 (100.00), 136 (6.51), 133 (5.46), 132 (34.79), 104 (19.95), 103 (7.02), 95 (27.88), 90 (5.27), 80 (25.66), 79 (5.02), 78 (6.35), 77 (26.54), 76 (12.05), 64 (26.21), 52 (17.43), 51 (28.02), 50 (13.37); Anal. Calcd for C_15_H_11_N_3_O_4_: C, 60.61; H, 3.73; N, 14.14%; found: C, 60.55; H, 3.78; N, 14.00%.

#### 3.2.4. General Method for the Synthesis of the Acetylated Acyclic Aromatic C-Nucleosides **5** and **6**

A solution of 1',2',3',4'-tetrahydroxybutyl derivatives **3b** and **3e** (2.544 mmoL) in dry pyridine (10 mL) was treated with acetic anhydride (10 mL), and the mixture was kept at room temperature for 5–12 h with occasional shaking. Then it was poured onto crushed ice, the acetyl derivative that separated out, was filtered off, washed with water and dried.

*N-(4-Nitrobenzylidene)-5-(1',2',3',4'-tetraacetoxybutyl)-2-methylfuran-3-carbohydrazide* (**5**). Obtained in 95% yield from compound **3b** (2.544 mmoL); recrystallized from methanol as yellow crystals; Rf: 0.67 (*n*-hexane–ethyl acetate, 1:1, *v/v*); mp 158–159 °C; IR (KBr): 3286 (NH), 1741 (OAc), 1648 (CO-amide), 1580 (C=N); ^1^H-NMR (CDCl_3_); δ: 1.95, 2.03 and 2.04 (3s, 12H, 4OAc), 2.51 (s, 3H, CH_3(furan)_), 4.08–4.12 (dd, 1H, H-4'b, *J*_3',4'b_ = 5.35 Hz, *J*_4'b,4'a_ = 12.20 Hz), 4.18 (d, 1H, H-4'a, *J*_4'b,4'a_ = 11.50 Hz), 5.05–5.06 (m, 1H, H-3'), 5.44 (bs, 1H, H-2'), 5.99 (d, 1H, H-1', *J*_1',2'_ = 4.6 Hz), 6.98 (s, 1H, CH_(furan)_), 7.93 (d, 2H, Ar-H_(b)_, *J* = 7.65 Hz), 8.26 (d, 2H, Ar-H_(a)_, *J* = 7.65 Hz), 8.43 (s, 1H, CH=N), 11.69 (s, 1H, NH, D_2_O exchangeable); MS: *m/z* (%), 562 (10.89, M^+^+1), 561 (15.45, M^+^), 546 (16.04), 520 (20.79), 515 (14.46), 489 (10.30), 439 (11.88), 417 (15.64), 397 (11.88), 385 (18.61), 345 (10.89), 324 (11.88), 273 (10.30), 265 (20.99), 252 (10.89), 227 (10.69), 180 (10.69), 109 (17.62), 108 (15.45), 94 (15.64), 81 (46.73), 80 (18.22), 72 (17.62), 69 (100.00), 66 (10.69), 65 (11.49), 57 (54.65), 55 (49.50); Anal. Calcd for C_25_H_27_N_3_O_12_: C, 53.48; H, 4.85; N, 7.48%; found: C, 53.40; H, 4.71; N, 7.50%.

*5-(1',2',3',4'-Tetraacetoxybutyl)-2-methyl-N-(N'-acetyl-2-oxoindolin-3-ylidene)furan-3-carbohydrazide* (**6**). Obtained in 100% yield from compound **3e**; recrystallized from ethanol as yellow needles; mp 154–155 °C; Rf: 0.54 (*n*-hexane–ethyl acetate; 5:1; *v/v*); IR (KBr) cm^−1^: 3283 (NH), 1748 (OAc), 1712 (2CO & NAc), 1608 (C=N); ^1^H-NMR (CDCl_3_); δ: 2.03, 2.05, 2.10 (3s, 12H, 4OAc), 2.65 (s, 3H, CH_3(furan)_), 2.73 (s, 3H, N-Ac), 4.10–4.16 (dd, 1H, H-4'b, *J*_4'a,4'b_ = 12.00 Hz, *J*_4'b,3'_ = 3.00 Hz), 4.23–4.28 (dd, 1H, H-4'a, *J*_4'a,4'b_ = 12.00 Hz, *J*_4'a,3'_ = 3.00 Hz), 5.16–5.21 (m, 1H, H-3'), 5.60–5.64 (dd, 1H, H-2', *J*_1',2'_ = 3.00 Hz, *J*_2',3'_ = 6.00 Hz), 6.08 (d, 1H, H-1', *J*_1',2'_ = 3.00 Hz), 6.70 (s, 1H, CH_(furan)_), 7.27 (t, 1H, Ar-H_(d)_, *J* = 9.00 Hz), 7.42 (t, 1H, Ar-H_(c)_, *J* = 9.00 Hz), 7.82 (d, 1H, Ar-H_(b)_, *J* = 9.00 Hz), 8.23 (d, 1H, Ar-H_(a)_, *J* = 9.00 Hz), 13.01 (bs, 1H, NH, D_2_O exchangeable); MS: *m/z* (%), 601 (16.75, M^+^+2), 600 (14.35, M^+^+1), 599 (18.90, M^+^),567 (22.73), 562 (22.49), 556 (14.35), 540 (17.70), 525 (22.49), 518 (21.53), 513 (13.88), 511 (21.29), 490 (18.66), 469 (18.66), 454 (17.70), 452 (23.92), 439 (23.21), 403 (20.10), 397 (52.63), 381 (14.83), 353 (26.56), 340 (26.56), 335 (19.38), 302 (27.27), 272 (21.29), 234 (25.84), 202 (30.14), 193 (92.82), 175 (38.52), 166 (21.53), 151 (32.30), 137 (100.00), 124 (26.56), 115 (28.23), 110 (39.23), 95 (33.97), 77 (42.34), 55 (28.47), 53 (15.55); Anal. Calcd for C_28_H_29_N_3_O_12_: C, 56.09; H, 4.88; N, 7.01%; found: C, 56.03; H, 4.92; N, 6.89%.

*1'-[(5-Methyl-4-(5-(4-nitrophenyl)-1,3,4-oxadiazol-2-yl)furan-2-yl)]butane-1',2',3',4'-tetrayl tetraacetate* (**7**). A solution of compound **5** (5.523 mmol) in dry ether (75 mL) was stirred with yellow mercuric oxide (4.80 g), magnesium oxide (0.48 g), and iodine (4.00 g) at room temperature for 48 h under anhydrous conditions. The reaction mixture was filtered off, and the filtrate washed with potassium iodide solution, sodium thiosulphate, and water respectively, and dried over anhydrous sodium sulphate. On evaporation of the dried filtrate, a yellow crystalline mass was obtained. An additional crop was obtained by extracting the inorganic residue with chloroform which upon concentration yielded the same product. Yield (50%); recrystallized from methanol as yellow crystals; Rf: 0.74 (*n*-hexane–ethyl acetate; 2:1; *v/v*); mp 147–148 °C; IR (KBr) cm^−1^: 1750 (OAc), 1635 (C=N); ^1^H-NMR (CDCl_3_); δ: 1.96, 1.97, 2.05, 2.06 (4s, 12H, 4OAc), 2.65 (s, 3H, CH_3(furan)_), 4.11–4.14 (dd, 1H, H-4'b, *J*_4'a,4'b_ = 12.20 Hz, *J*_4'b,3'_ = 5.35 Hz), 4.20–4.23 (dd, 1H, H-4'a, *J*_4'a,4'b_ = 12.20 Hz, *J*_4'a,3'_ = 3.05 Hz), 5.08–5.11 (m, 1H, H-3'), 5.46–5.49 (dd, 1H, H-2', *J*_1',2'_ = 5.35 Hz, *J*_2',3'_ = 6.90 Hz), 6.05 (d, 1H, H-1', *J*_1',2'_ = 4.60 Hz), 7.03 (s, 1H, CH_(furan)_), 8.29 (d, 2H, Ar-H_(b)_, *J* = 8.45 Hz), 8.40 (d, 2H, Ar-H_(a)_, *J* = 9.15 Hz); MS: *m/z* (%), 561 (13.74, M^+^+2), 560 (23.92, M^+^+1), 559 (18.83), 549 (19.34), 528 (21.88), 471 (20.10), 462 (20.61), 457 (29.52), 378 (20.87), 368 (19.34), 301 (25.45), 289 (20.10), 270 (24.68), 250 (24.94), 209 (21.88), 185 (21.37), 183 (21.88), 138 (21.37), 119 (21.37), 82 (23.41), 75 (19.85), 64 (23.41), 63 (17.81), 60 (100.00), 55 (23.92), 57 (22.14); Anal. Calcd for C_25_H_25_N_3_O_12_: C, 53.67; H, 4.50; N, 7.51%; found: C, 53.71; H, 4.44; N, 7.44%.

*1'-[(5-Methyl-4-(5-(4-nitrophenyl)-1,3,4-oxadiazol-2-yl)furan-2-yl)]butane-1',2',3',4'-tetraol* (**8**). A solution of compound **7** (0.894 mmoL) was heated with hydrazine hydrate (10 mL) in methanol (10 mL) under reflux for 1 h. The 1',2',3',4'-tetraol **8** that separated out was filtered off, washed with methanol and dried. Yield 97%; recrystallized from ethanol as pale yellow crystals; mp 147 °C; Rf: 0.62 (chloroform–methanol; 5:1; *v/v*); IR (KBr) cm^−1^: 3307 (OH), 1646 (C=N); ^1^H-NMR (DMSO-*d*_6_); δ: 2.64 (s, 3H, CH_3(furan)_), 3.39–3.43 (m, 1H, H-4'b), 3.47–3.52 (m, 1H, H-3'), 3.54–3.56 (m, 1H, H-2'), 3.57–3.61 (m, 1H, H-4'a), 4.37 (t, 1H, 4'-OH, *J*_4',OH_ = 6.10 Hz, D_2_O-exchangeable), 4.62 (d, 1H, 3'-OH, *J*_3',OH_ = 6.90 Hz, D_2_O-exchangeable), 4.66 (d, 1H, 2'-OH, *J*_2',OH_ = 5.35 Hz, D_2_O-exchangeable), 4.81 (d, 1H, H-1', *J*_1',2'_ = 6.85 Hz), 5.23 (d, 1H, 1'-OH, *J*_1',OH_ = 7.65 Hz, D_2_O-exchangeable), 6.73 (s, 1H, CH_(furan)_), 8.27 (d, 2H, Ar-H_(b)_, *J* = 8.40 Hz), 8.39 (d, 2H, Ar-H_(a)_, *J* = 8.40 Hz); MS: *m/z* (%), 393 (28.16, M^+^+2), 392 (30.58, M^+^+1), 391 (37.86, M^+^), 356 (43.20), 307 (48.54), 300 (100.00), 295 (39.81), 284 (49.51), 279 (44.66), 237 (40.78), 236 (41.75), 228 (40.78), 222 (42.23), 154 (57.77), 147 (43.69), 144 (45.63), 139 (30.58), 137 (75.73), 127 (38.35), 120 (39.32), 104 (67.96), 95 (46.12), 90 (49.51), 79 (43.69), 76 (94.17), 75 (61.65), 57 (48.54), 56 (93.69), 50 (60.19); Anal. Calcd for C_17_H_17_N_3_O_8_: C, 52.18; H, 4.38; N, 10.74%; found: C, 52.24; H, 4.17; N, 10.76%.

*Tetrahydro-1'-[(5-methyl-4-(5-(4-nitrophenyl)-1,3,4-oxadiazol-2-yl)furan-2-yl)]-furan-2',3'-diol* (**9**). A solution of compound **8** (0.767 mmoL) was heated with aqueous acetic acid (150 mL, 10%) under reflux for 8 h After cooling the 2',3'-diol **9** that separated out was filtered off, washed with water and dried. Yield 63%; recrystallized from ethanol as a yellow powder; mp 199–200 °C; Rf: 0.88 (chloroform–methanol; 5:1; *v/v*); IR (KBr) cm^−1^: 3372 (OH), 1637 (C=N); ^1^H-NMR (DMSO-*d*_6_); δ: 2.66 (s, 3H, CH_3(furan)_), 3.62–3.64 (dd, 1H, H-4'b, *J*_4'b,3'_ = 2.30 Hz, *J*_4'b,4'a_ = 9.15 Hz), 4.02–4.05 (dd, 1H, H-4'a, *J*_3',4'a_ = 4.55 Hz, *J*_4'b,4'a_ = 9.20 Hz), 4.10–4.17 (m, 2H, H-3', H-2'), 4.54 (d, 1H, H-1', *J*_1',2'_ = 7.60 Hz), 5.03 (d, 1H, 3'-OH, *J*_3',OH_ = 3.85 Hz, D_2_O-exchangeable), 5.16 (d, 1H, 2'-OH, *J*_2',OH_ = 6.1 Hz, D_2_O-exchangeable), 6.93 (s, 1H, CH_(furan)_), 8.28 (d, 2H, Ar-H_(b)_, *J* = 8.40 Hz), 8.39 (d, 2H, Ar-H_(a)_,*J* = 9.20 Hz); MS: *m/z* (%), 374 (41.04, M^+^+1), 373 (42.54, M^+^), 328 (48.51), 327 (52.99), 284 (64.93), 257 (79.10), 254 (54.48), 247 (56.72), 238 (67.16), 211 (52.99), 208 (50.75), 206 (50.75), 170 (66.42), 165 (54.48), 154 (52.99), 148 (62.69), 127 (72.39), 109 (52.24), 104 (73.13), 103 (58.96), 77 (56.72), 76 (100.00), 75 (58.96), 65 (92.54), 64 (60.45), 56 (62.69), 50 (47.01); Anal. Calcd for C_17_H_15_N_3_O_7_: C, 54.69; H, 4.05; N, 11.26%; found: C, 54.70; H, 4.00; N, 11.21%.

*4-(9b-Acetyl-5,9b-dihydro-5-oxo-[1,3,4]oxadiazolo[2,3-a]isoindol-2-yl)-5-methylfuran-2-yl acetate* (**10b**). A solution of compound **3a** (0.765 mmoL) was boiled with acetic anhydride (4 mL) in the presence of anhydrous sodium acetate (0.25 g) for 7 h. The acetyl derivative that separated was filtered off and dried. Yield 59%; recrystallized from methanol as colourless crystals; Rf: 0.47 (*n*-hexane-ethyl acetate, 4:1, *v/v*); mp 168–169 °C; IR (KBr) cm^−1^: 1744 (OAc), 1725 (COCH_3_), 1712 (CO-isoindolone), 1618 (C=N); ^1^H-NMR (CDCl_3_); δ: 2.14 (s, 3H, OAc), 2.27 (s, 3H, COCH_3_), 2.56 (s, 3H, CH_3(furan)_), 7.00 (s, 1H, CH_(furan)_), 7.53 (d, 1H, Ar-H_(d)_, *J* = 7.65 Hz), 7.56 (t, 1H, Ar-H_(c)_, *J* = 7.65 Hz), 7.65 (t, 1H, Ar-H_(b)_, *J* = 7.65 Hz), 7.84 (d, 1H, Ar-H_(a)_, *J* = 7.65 Hz); MS: *m/z* (%), 356 (2.50, M^+^+2), 355 (2.33, M^+^+1), 354 (3.54, M^+^), 248 (9.62), 189 (15.92), 188 (31.58), 148 (10.09), 147 (23.86), 146 (100.00), 133 (29.25), 118 (10.27), 105 (24.07), 104 (10.66), 89 (33.82), 77 (24.16), 76 (16.31), 63 (12.12), 60 (9.15), 51 (13.03); Anal. Calcd for C_18_H_14_N_2_O_6_: C, 61.02; H, 3.98; N, 7.91%; found: C, 61.00; H, 4.00; N, 7.99%.

#### 3.2.5. General Procedures for the Preparation of the Aromatic C-Nucleosides **12**–**14**

*Method A*. A solution of compounds **3b** and **3e** (2.544 mmoL) was heated with aqueous acetic acid (150 mL, 10%) under reflux for 5 h. After cooling the 3-carbohydrazones **12**, **13** that separated out were filtered off, washed with water and dried.

*Method B*. A solution of 5-(2',3'-dihydroxytetrahydrofuran-1'-yl)-2-methylfuran-3-carbohydrazide (**11**) [4] in ethanol containing a few drops of acetic acid was treated with carbonyl compound, and the reaction mixture was refluxed on water bath for 30 min. After cooling the 3-carbohydrazones **12**–**14** that separated out were filtered off, washed with a little ethanol, and dried.

*N-(4-Nitrobenzylidene)-5-(tetrahydro-2',3'-dihydroxyfuran-1'-yl)-2-methylfuran-3-carbohydrazide* (**12**). Obtained in 90% yield from **11** (0.413 mmol) and 4-nitrobenzaldehyde (0.413 mmol); recrystallized from ethanol as yellow needles; mp 239–240 °C; Rf: 0.69 (chloroform–methanol; 5:1; *v/v*); IR(KBr) cm^−1^: 3292–3245 (OH, NH), 1660 (CO-amide), 1614 (C=N); ^1^H-NMR (DMSO-*d*_6_); δ: 2.52 (s, 3H, CH_3(furan)_), 3.61–3.63 (dd, 1H, H-4'b, *J*_3',4'b_ = 2.30 Hz, *J*_4'b,4'a_ = 9.20 Hz), 4.01–4.04 (dd, 1H, H-4'a, *J*_3',4'a_ = 4.60 Hz, *J*_4'b,4'a_ = 9.20 Hz), 4.06–4.11(m, 2H, H-3', H-2'), 4.49 (d, 1H, H-1', *J*_1',2'_ = 6.85 Hz), 5.02 (d, 1H, 3'-OH, *J_2',OH_* = 3.80 Hz, D_2_O-exchangeable), 5.12 (d, 1H, 2'-OH, *J_1',OH_* = 6.10 Hz, D_2_O-exchangeable), 6.88 (s, 1H, CH_(furan)_), 7.93 (d, 2H, Ar-H_(b)_, *J* = 8.45 Hz), 8.26 (d, 2H, Ar-H_(a)_, *J* = 8.45 Hz Hz), 8.44 (s, 1H, CH=N), 11.67 (s, 1H, NH, D_2_O-exchangeable); MS: *m/z* (%), 376 (4.69, M^+^+1), 375 (11.01, M^+^), 317 (7.58), 212 (12.18), 211 (100.00), 153 (7.94), 151 (51.35), 137 (12.55), 123 (16.52), 115 (9.03), 105 (8.30), 95 (11.10), 81 (13.63), 79 (14.53), 77 (4.15), 70 (9.75), 63 (8.03), 61 (12.36), 55 (12.82), 53 (10.29), 52 (11.82); Anal. Calcd for C_17_H_17_N_3_O_7_: C, 54.40; H, 4.57; N, 11.20%; found: C, 54.50; H, 4.60; N, 11.11%.

*5-(Tetrahydro-2',3'-dihydroxyfuran-1'-yl)-2-methyl-N-(2-oxoindolin-3-ylidene)furan-3-carbohydrazide* (**13**). Obtained in 91% yield from **11** (0.413 mmol) and isatin (0.413 mmol); recrystallized from ethanol as yellow needles; mp 263–264 °C; Rf: 0.69 (chloroform–methanol; 5:1; *v/v*); IR (KBr) cm^−1^: 3420 (OH), 3166 (2NH), 1703 (CO-oxoindoline), 1673 (CO-amide), 1619 (C=N); ^1^H-NMR (DMSO-*d*_6_); δ: 2.57 (s, 3H, CH_3(furan)_), 3.62 (d, 2H, H-4'b, *J*_4'b,4'a_ = 9.00 Hz), 4.01–4.06 (dd, 1H, H-4'a, *J*_3',4'a_ = 6.00 Hz, *J*_4'b,4'a_ = 9.00 Hz), 4.10 (bs, 2H, H-3', H-2'), 4.53 (d, 1H, H-1', *J*_1',2'_ = 9.00 Hz), 4.94 (bs, 1H, 3'-OH, D_2_O-exchangeable), 5.09 (bs, 1H, 2'-OH, D_2_O-exchangeable), 6.64 (s, 1H, CH_(furan)_), 6.93 (d, 1H, Ar-H_(d)_, *J* = 9.00 Hz), 7.07 (t, 1H, Ar-H_(c)_, *J* = 9.00 Hz), 7.35 (t, 1H, Ar-H_(b)_, *J* = 9.00 Hz), 7.55 (d, 1H, Ar-H_(a)_, *J* = 9.00 Hz), 11.25 (bs, 1H, NH_(2)_, D_2_O-exchangeable), 13.35 (bs, 1H, NH_(1)_, D_2_O-exchangeable); MS: *m/z* (%), 372 (33.14, M^+^+1), 371 (44.57, M^+^), 340 (37.14), 335 (37.14), 294 (40.00), 286 (49.14), 276 (40.00), 268 (37.14), 187 (40.57), 167 (44.57), 141 (40.57), 138 (40.00), 135 (49.71), 126 (43.43), 125 (44.57), 94 (100.00), 88 (46.86), 87 (49.71), 85 (46.86), 83 (55.43), 81 (62.86), 80 (77.14), 79 (48.00), 77 (45.14), 73 (66.29), 72 (42.29), 71 (60.00), 69 (85.71), 64 (69.14), 61 (43.43), 60 (65.14), 57 (82.86), 55 (72.57); Anal. Calcd for C_18_H_17_N_3_O_6_: C, 58.22; H, 4.61; N, 11.32%; found: C, 58.15; H, 4.65; N, 11.44%.

*5-(Tetrahydro-2',3'-dihydroxyfuran-1'-yl)-N-2,3,4,5,6-pentahydroxyhexylidene)-2-methylfuran-3-carbohydrazide* (**14**). Obtained in 93% yield from **11** (0.413 mmol) and d-galactose (0.826 mmol); recrystallized from ethanol as pale yellow syrup; Rf: 0.44 (chloroform–methanol, 1:1, *v/v*); IR (KBr) cm^−1^: 3380–3150 (OH, NH), 1648 (CO), 1612 (C=N); MS: *m/z* (%), 406 (18.88, M^+^+2), 405 (23.08, M^+^+1), 404 (19.93, M^+^), 395 (24.83), 363 (26.57), 362 (25.87), 354 (23.78), 313 (25.52), 305 (24.48), 294 (23.78), 282 (24.48), 262 (28.67), 250 (26.57), 236 (24.48), 235 (28.67), 228 (28.67), 225 (23.78), 219 (28.67), 209 (24.83), 208 (30.07), 205 (25.87), 175 (25.52), 173 (25.52), 158 (24.83), 157 (26.57), 122 (27.62), 107 (28.67), 73 (100.00), 71 (39.16), 61 (40.91), 60 (81.82), 56 (26.57), 52 (26.57).

#### 3.2.6. General Procedure for the Acetylation of the Aromatic C-Nucleosides **12** and **13**

A solution of 2',3'-dihydroxyfurans **12** and **13** (0.809 mmoL) in dry pyridine (15 mL) was treated with acetic anhydride (15 mL) and the mixture was kept overnight with occasional shaking at room temperature. Then it was poured onto crushed ice, the acetyl derivatives **15** and **16** that separated out were filtered off, washed with water and dried.

*N-(4-Nitrobenzylidene)-5-(tetrahydro-2',3'-diacetoxyfuran-1'-yl)-2-methylfuran-3-carbohydrazide* (**15**). Obtained in 79% yield from **12** (0.809 mmoL); recrystallized from ethanol as yellow crystals; mp 173–174 °C; Rf: 0.61 (*n*-hexane–ethyl acetate; 1:1; *v/v*); IR (KBr) cm^−1^: 3235 (NH), 1753 (OAc), 1654 (CO), 1600 (C=N); ^1^H-NMR (CDCl_3_); δ: 2.00, 2.06 (2s, 6H, 2OAc), 2.52 (s, 3H, CH_3(furan)_), 3.81–3.84 (dd, 1H, H-4'b, *J*_4'a,4'b_ = 10.70 Hz, *J*_4'b,3'_ = 2.30 Hz), 4.25–4.29 (dd, 1H, H-4'a, *J*_4'a,4'b_ = 10.70 Hz, *J*_4'a,3'_ = 5.35 Hz), 4.88 (d, 1H, H-3', *J*_2',3'_ = 6.10 Hz), 5.34–5.37 (m, 1H, H-2'), 5.40–5.42 (m, 1H, H-1'), 6.98 (s, 1H, CH_(furan)_), 7.93 (d, 2H, Ar-H_(b)_, *J* = 6.85 Hz), 8.26 (d, 2H, Ar-H_(a)_, *J* = 7.65 Hz), 8.43 (s, 1H, CH=N), 11.72 (s, 1H, NH, D_2_O exchangeable); MS: *m/z* (%), 460 (12.29, M^+^+1), 459 (16.74, M^+^), 400 (28.39), 380 (24.15), 340 (74.79), 323 (19.49), 296 (25.21), 295 (100.00), 265 (19.92), 260 (15.47), 258 (17.16), 235 (19.92), 193 (26.91), 192 (25.21), 175 (35.17), 151 (44.28), 150 (26.69), 147 (24.58), 138 (25.00), 137 (54.03), 123 (28.39), 115 (79.87), 105 (27.54), 95 (40.89), 91 (26.27), 85 (25.64), 27 (23.31), 76 (23.52), 63 (25.64), 55 (27.54), 52 (28.39); Anal. Calcd for C_21_H_21_N_3_O_9_: C, 54.90; H, 4.61; N, 9.15%; found: C, 54.79; H, 4.69; N, 9.24%.

*5-(Tetrahydro-2',3'-diacetoxyfuran-1'-yl)-2-methyl-N-(N'-acetyl-2-oxoindolin-3-ylidene)furan-3-carbohydrazide* (**16**). Obtained in 67% yield from **13** (0.809 mmoL); recrystallized from ethanol as yellow crystals; mp 162–163 °C; Rf: 0.51 (*n*-hexane–ethyl acetate; 5:1; *v/v*); IR (KBr) cm^−1^: 3273 (NH), 1745 (OAc), 1708 (3CO), 1603 (C=N); ^1^H-NMR (CDCl_3_); δ: 2.06, 2.11 (2s, 6H, 2OAc), 2.67 (s, 3H, CH_3(furan)_), 2.73 (s, 3H, N-Ac), 3.93–3.98 (dd, 1H, H-4'b, *J*_4'a,4'b_ = 12.00 Hz, *J*_4'b,3'_ = 3.00 Hz), 4.36–4.41 (dd, 1H, H-4'a, *J*_4'a,4'b_ = 12.00 Hz, *J*_4'a,3'_ = 3.00 Hz), 4.93 (d, 1H, H-3', *J*_2',3'_ = 6.00 Hz), 5.48–5.55 (m, 2H, H-2', H-1'), 6.74 (bs, 1H, CH_(furan)_), 7.27 (t, 1H, Ar-H_(d)_, *J* = 9.00 Hz), 7.42 (t, 1H, Ar-H_(c)_, *J* = 9.00 Hz), 7.82 (d, 1H, Ar-H_(b)_, *J* = 6.00 Hz), 8.23 (d, 1H, Ar-H_(a)_, *J* = 9.00 Hz), 13.03 (bs, 1H, NH, D_2_O exchangeable); MS: *m/z* (%), 499 (5.52, M^+^+2), 498 (7.88, M^+^+1), 496 (4.73, M^+^-1), 434 (8.23), 379 (11.03), 378 (31.35), 296 (21.54), 295 (100.00), 235 (10.16), 193 (13.66), 192 (10.42), 175 (18.83), 151 (13.84), 149 (13.40), 148 (11.30), 147 (14.19), 137 (34.68), 121 (16.20), 115 (40.72), 109 (13.84), 105 (17.86), 104 (17.43), 95 (34.85), 80 (14.89), 79 (15.06), 77 (23.64), 76 (11.56), 55 (13.22), 52 (12.00), 51 (14.36); Anal. Calcd for C_24_H_23_N_3_O_9_: C, 57.95; H, 4.66; N, 8.45%; found: C, 57.88; H, 4.52; N, 8.50%.

*5-(Tetrahydroj-2,2-dimethylfuro[2',3'-d][1,3]dioxol-1'-yl)-2-methyl-N-(2-oxoindolin-3-ylid-ene)furan-3-carbohydrazide* (**17**). Compound **13** (0.485 mmol) was treated with FeCl_3_ (0.035 g) in dry acetone (30 mL). The reaction mixture was heated under reflux for 30 min. After cooling it was poured onto cold water, the separated yellow crystals **17** was filtered off and dried. Yield 88%; recrystallized from ethanol as yellow needles; mp 220 °C; Rf: 0.75 (chloroform–methanol; 20:1; *v/v*); IR (KBr) cm^−1^: 3178 (2NH), 1684 (2CO), 1615 (C=N); ^1^H-NMR (DMSO-*d*_6_); δ: 1.26, 1.39 (2s, 6H, CMe_2_, Δδ = 0.13), 2.54 (s, 3H, CH_3(furan)_), 3.74–3.69 (dd, 1H, H-4'b, *J*_4'a,4'b_ = 12.00 Hz, *J*_4'b,3'_ = 3.00 Hz), 3.87 (d, 1H, H-4'a, *J*_4'a,4'b_ = 12.00 Hz), 4.88–4.95 (m, 2H, H-3', H-2'), 4.98 (s, 1H, H-1', *J*_1',2'_ = 0 Hz ), 6.63 (bs, 1H, CH_(furan)_), 6.92 (d, 1H, Ar-H_(d)_, *J* = 9.00 Hz), 7.06 (t, 1H, Ar-H_(c)_, *J* = 9.00 Hz), 7.36 (t, 1H, Ar-H_(b)_, *J* = 6.00 Hz), 7.53 (d, 1H, Ar-H_(a)_, *J* = 9.00 Hz), 11.28 (s, 1H, NH_(2)_, D_2_O exchangeable), 13.35 (bs, 1H, NH_(1)_, D_2_O exchangeable); MS: *m/z* (%), 413 (0.88, M^+^+2), 412 (5.26, M^+^+1), 411 (20.24, M^+^), 383 (9.26), 252 (16.46), 251 (100.00), 160 (24.84), 159 (10.29), 151 (8.40), 137 (23.36), 132 (16.47), 123 (6.97), 121 (12.88), 110 (8.02), 109 (6.21), 105 (6.89), 104 (15.66), 95 (14.58), 80 (10.20), 79 (18.26), 78 (6.11), 77 (22.12), 76 (6.12), 69 (7.01), 65 (6.34), 59 (14.57), 57 (6.78), 55 (12.70), 53 (8.67), 52 (10.78), 51 (11.09); Anal. Calcd for C_21_H_21_N_3_O_6_: C, 61.31; H, 5.14; N, 10.21%; found: C, 61.26; H, 5.19; N, 10.18%.

*(5-(1',2',3',4'-Tetrahydroxybutyl)-2-methylfuran-3-yl)(3,5-dimethyl-1H-pyrazol-1-yl)-methanone* (**18**). A mixture of 3-carbohydrazide **1** (5 mmoL) and acetylacetone (5 mmoL) was heated under reflux in ethanol (10 mL) containing a few drops of acetic acid for 5 h. After cooling the solid **18** that separated was filtered off and dried. Yield 100%; recrystallized from ethanol as colorless needles; mp 142–143 °C; Rf: 0.55 (chloroform–methanol; 5:1; *v/v*); IR (KBr) cm^−1^: 3314 (OH), 1689 (CO), 1560 (C=N); ^1^H-NMR (DMSO-*d*_6_); δ: 2.16 (s, 3H, CH_3(b-pyrazole)_), 2.48 (s, 6H, CH_3(furan)_ and CH_3(a-pyrazole)_), 3.39–3.43 (m, 1H, H-4'b), 3.47–3.58 (m, 3H, H-3', H-2', H-4'a), 4.30 (bs, 1H, 4'-OH, D_2_O-exchangeable), 4.48 (d, 1H, 3'-OH, *J_3',OH_* = 6.00 Hz, D_2_O-exchangeable), 4.56 (bs, 1H, 2'-OH, D_2_O-exchangeable), 4.76 (d, 1H, H-1', *J*_1',2'_ = 6.00 Hz), 5.11 (d, 1H, 1'-OH, *J*_1',OH_ = 6.00 Hz, D_2_O-exchangeable), 6.18 (s, 1H, CH_(pyrazole)_), 6.90 (s, 1H, CH_(furan)_); MS: *m/z* (%), 325 (0.17, M^+^+1), 324 (0.30, M^+^), 306 (17.49), 234 (9.75), 233 (32.42), 211 (26.86), 210 (75.30), 203 (23.37), 182 (23.85), 151 (6.04), 138 (6.24), 137 (41.72), 123 (6.09), 122 (7.28), 121 (12.20), 110 (13.35), 109 (12.02), 103 (8.55), 97 (100.00), 96 (10.91), 95 (18.16) 81 (11.08), 79 (11.69), 56 (6.59), 55 (9.48), 53 (11.53), 51 (7.48); Anal. Calcd for C_15_H_20_N_2_O_6_: C, 55.55; H, 6.22; N, 8.64%; found: C, 55.56; H, 6.20; N, 8.59%.

*(5-(1',2',3',4'-Tetraacetoxybutyl)-2-methylfuran-3-yl)(3,5-dimethyl-1H-pyrazol-1-yl)methanone* (**19**). A solution of compound **18** (1.543 mmoL) in dry pyridine (15 mL) was treated with acetic anhydride (15 mL) and the mixture was kept overnight with occasional shaking at room temperature. Then it was poured onto crushed ice and the acetyl derivative **19** that separated out was filtered off, washed with water and dried. Yield 68%; recrystallized from ethanol as colorless needles; mp 101–102 °C; Rf: 0.74 (*n*-hexane–ethyl acetate; 2:1; *v/v*); IR (KBr) cm^−1^: 1751 (OAc), 1683 (CO), 1591 (C=N); ^1^H-NMR (CDCl_3_); δ: 2.03, 2.04, 2.06, 2.12 (4s, 12H, 4OAc), 2.23 (s, 3H, CH_3(pyrazole-b)_), 2.54 (s, 3H, CH_3(furan)_), 2.57 (s, 3H, CH_3(pyrazole-a)_), 4.09–4.14 (dd, 1H, H-4'b, *J*_4'a,4'b_ = 12.00 Hz, *J*_4'b,3'_ = 3.00 Hz), 4.19–4.24 (dd, 1H, H-4'a, *J*_4'a,4'b_ = 12.00 Hz, *J*_4'a,3'_ = 3.00 Hz), 5.14–5.19 (m, 1H, H-3'), 5.60–5.64 (dd, 1H, H-2',*J*_1',2'_ = 3.00 Hz, *J*_2',3'_ = 6.00 Hz), 5.97 (s, 1H, CH_(pyrazole)_), 6.08 (d, 1H, H-1', *J*_1',2'_ = 3.00 Hz), 7.15 (s, 1H, CH_(furan)_); ^13^C-NMR (CDCl_3_); δ: 13.86, 14.47 and 14.58 for (2CH_3(pyrazole)_, CH_3(furan)_), 20.74, 20.80 (2 lines for 4 OCOCH_3_), 61.65, 65.70, 68.66 and 69.86 for (C-4', C-3', C-2' and C-1'), 110.97, 113.77, 116.00, 144.72, 145.84, 151.87, 162.89 for (pyrazole and furan carbons), 169.50 (CO-N), 169.70, 169.80, 169.95, 170.60 (4 OCOCH_3_); MS: *m/z* (%), 494 (4.19, M^+^+2), 493 (7.52, M^+^+1), 492 (18.80, M^+^), 433 (9.49), 432 (10.73), 397 (13.56), 396 (27.19), 373 (11.47), 372 (9.25), 336 (27.99), 330 (11.96), 275 (10.91), 235 (16.40), 234 (60.30), 233 (86.68), 203 (33.48), 193 (19.36), 192 (30.83), 180 (09.00), 179 (10.11), 175 (12.08), 151 (10.97), 150 (09.25), 138 (19.79), 137 (75.71), 136 (09.49), 123 (09.12), 122 (10.42), 121 (13.69), 115 (23.06), 110 (19.30), 109 (17.02), 103 (09.99), 98 (10.60), 97 (100.00), 96 (17.32), 95 (25.77), 83 (10.42), 81 (16.83), 80 (10.79), 79 (15.84), 73 (11.59), 71 (11.41), 69 (16.71), 67 (10.23), 61 (10.48), 57 (19.17), 55 (22.32), 54 (09.80), 53 (11.47), 51 (10.30); Anal. Calcd for C_23_H_28_N_2_O_10_: C, 56.09; H, 5.73; N, 5.69%; found: C, 56.00; H, 5.62; N, 5.77%.

1-(5-(1',2',3',4'-Tetrahydroxybutyl)-2-methylfuran-3-carbo-3-yl)-4-phenyl thiosemicarbazide (**20**) [24]. A mixture of 5-(1',2',3',4'-tetrahydroxybutyl)-2-methylfuran-3-carbohydrazide **1** (3.846 mmoL) and phenyl isothiocyanate (3.846 mmoL) are heated under reflux in ethanol (10 mL) for 2 h. After cooling the thiosemicarbazide that separated was filtered off, washed with little ethanol, and dried. Yield (100%); recrystallized from ethanol as white crystals; mp 133–134 °C; Rf: 0.35 (chloroform–methanol; 5:1; *v/v*); IR(KBr) cm^−1^: 3415–3264 (OH, 3NH), 1659 (CO); ^1^H-NMR (DMSO-*d*_6_); δ: 2.47 (s, 1H, CH_3_-furan with DMSO), 3.34–3.41 (m, 1H, H-4'b), 3.45–3.48 (m, 1H, H-4'a), 3.50–3.57 (m, 2H, H-3', H-2'), 4.34 (t, 1H, 4'-OH, *J*_4',OH_ = 6.15 Hz, D_2_O-exchangeable), 4.42 (d, 1H, 3'-OH, *J*_3',OH_ = 6.85 Hz, D_2_O-exchangeable), 4.59 (d, 1H, 2'-OH, *J*_2',OH_ = 6.15 Hz, D_2_O-exchangeable), 4.73 (d, 1H, H-1', *J*_1',2'_ = 7.65 Hz), 5.12 (d, 1H, 1'-OH, *J*_1',OH_ = 8.40 Hz, D_2_O-exchangeable), 6.72 (s, 1H, CH _(furan)_), 7.11 (t, 1H, Ar-H_(f)_, *J* = 8.40 Hz), 7.28 (t, 2H, Ar-H_(e)_, *J* = 9.15 Hz), 7.42 (bs, 2H, Ar-H_(d)_), 9.54 (s, 1H, NH_(c)_, D_2_O exchangeable), 9.72 (s, 1H, NH_(b)_, D_2_O exchangeable), 9.97 (s, 1H, NH_(a)_, D_2_O exchangeable); Anal. Calcd for C_17_H_21_N_3_O_6_S: C, 51.64; H, 5.35; N, 10.63%; found: C, 51.70; H, 5.20; N, 10.60%.

*1'-(5-Methyl-4-(5-(phenylamino)-1,3,4-oxadiazol-2-yl)furan-2-yl)butane-1',2',3',4'-tetraol* (**21**). To a suspension of thiosemicarbazide **20** (5.063 mmol) in ethanol (50 mL), sodium hydroxide solution (4 N, 5 mL) was added with shaking. A solution of iodine and potassium iodide was added dropwise with stirring till the color of iodine persisted. The precipitate **21** was filtered off, washed with sodium thiosulphate solution, then with water, and dried. Yield (95%); recrystallized from ethanol as white crystals; mp 223–224 °C; Rf: 0.71 (chloroform–methanol; 4:1; *v/v*); IR(KBr) cm^−1^: 3421–3318 (OH), 3242 (NH), 1673 (C=N); ^1^H-NMR (DMSO-*d*_6_); δ: 2.54 (s, 3H, CH_3(furan)_), 3.41–3.55 (m, 4H, H-4'b, H-3', H-2', H-4'a,), 4.37 (t, 1H, 4'-OH, *J*_4',OH_ = 6.00 Hz, D_2_O-exchangeable), 4.62 (d, 1H, 3'-OH, *J*_3',OH_ = 9.00 Hz, D_2_O-exchangeable), 4.65 (d, 1H, 2'-OH, *J*_2',OH_ = 3.00 Hz, D_2_O-exchangeable), 4.77 (d, 1H, H-1', *J*_1',2'_ = 6.00 Hz), 5.19 (d, 1H, 1'-OH, *J*_1',OH_ = 6.00 Hz, D_2_O-exchangeable), 6.53 (s, 1H, CH_(furan)_), 6.96 (t, 1H, Ar-H_(c)_, *J* = 9.00 Hz), 7.32 (t, 2H, Ar-H_(b)_, *J* = 9.00 Hz), 7.55 (d, 2H, Ar-H_(a)_, *J* = 9.00 Hz), 10.50 (s, 1H, NH, D_2_O-exchangeable); MS: *m/z* (%), 361 (14.29, M^+^), 360 (27.07), 359 (18.30), 312 (19.05), 287 (17.04), 283 (26.32), 247 (19.80), 241 (17.04), 229 (177.79), 221 (23.06), 204 (18.55), 181 (17.04), 173 (20.30), 172 (17.54), 167 (17.54), 152 (20.55), 148 (16.54), 141 (21.05), 137 (15.54), 135 (24.56), 120 (21.80), 117 (18.30), 111 (27.57), 110 (19.05), 109 (20.30), 103 (19.80), 99 (21.55), 98 (26.57), 97 (35.84), 96 (31.08), 95 (30.58), 93 (25.56), 85 (34.59), 84 (29.07), 83 (51.13), 82 (24.31), 81 (37.59), 80 (39.60), 77 (26.32), 76 (18.55), 75 (17.79), 73 (29.82), 71 (49.62), 70 (32.58), 69 (67.67), 68 (29.57), 67 (38.60), 64 (28.57), 61 (19.05), 60 (39.60), 57 (100.00), 56 (27.57), 55 (93.98), 54 (30.58), 53 (26.32), 52 (19.80), 51 (23.06); Anal. Calcd for C_17_H_19_N_3_O_6_: C, 56.51; H, 5.30; N, 11.63%; found: C, 56.47; H, 5.41; N, 11.70%.

*1'-[5-Methyl-4-(5-(phenylamino)-1,3,4-oxadiazol-2-yl)furan-2-yl]butane-1',2',3',4'-tetrayl tetraacetate* (**22**). A solution of compound **21** (1.108 mmoL) in dry pyridine (10 mL) was treated with acetic anhydride (10 mL) and the mixture was kept overnight with occasional shaking at room temperature. Then it was poured onto crushed ice, and the acetyl derivative **22** that separated out was filtered off, washed with water and dried. Yield (85%); recrystallized from ethanol as white crystals; mp 180–181 °C; Rf: 0.5 (*n*-hexane–ethyl acetate; 2:1; *v/v*); IR(KBr) cm^−1^: 3136 (NH), 1747 (OAc), 1622 (C=N); ^1^H-NMR (CDCl_3_); δ: 2.04, 2.06, 2.08, 2.09 (4s, 12H, 4OAc), 2.60 (s, 3H, CH_3(furan)_), 4.09–4.15 (dd, 1H, H-4'b, *J*_4'a,4'b_ = 12.00 Hz, *J*_4'b,3'_ = 6.00 Hz), 4.22–4.27 (dd, 1H, H-4'a, *J*_4'a,4'b_ = 12.00 Hz, *J*_4'a,3'_ = 3.00 Hz), 5.16–5.21 (m, 1H, H-3'), 5.59–5.63 (dd, 1H, H-2', *J*_1',2'_ = 3.00 Hz, *J*_2',3'_ = 6.00 Hz), 6.06 (d, 1H, H-1', *J*_1',2'_ = 3.00 Hz), 6.69 (s, 1H, CH_(furan)_), 7.07 (t, 1H, Ar-H_(i)_*, J* = 9.00 Hz), 7.35 (t, 2H, Ar-H_(h)_, *J* = 9.00 Hz), 7.48 (d, 2H, Ar-H_(g)_, *J* = 9.00 Hz), 8.01 (bs, 1H, NH, D_2_O-exchangeable); ^13^C-NMR (CDCl_3_); δ: 13.72 (CH_3(furan)_), 20.72, 20.78, 20.84 (3 lines for 4 COCH_3_), 61.65 (C-4'), 65.86 (C-3'), 68.61 (C-2'), 69.79 (C-1'), 107.45 (j), 108.99 (i), 117.66 (h), 123.17 (g), 129.48 (f), 137.77 (e), 147.91 (d), 154.14 (c), 154.41 (b), 159.51 (a), 169.43, 169.74, 169.84, 170.62 (4 COCH_3_); MS: *m/z* (%), 531 (11.12, M^+^+2), 530 (6.24, M^+^+1), 529 (19.12, M^+^), 496 (10.05), 476 (8.68), 452 (9.46), 428 (11.12), 388 (8.39), 374 (8.98), 368 (21.56), 367 (34.93), 329 (10.34), 325 (28.88), 312 (11.41), 308 (16.29), 307 (10.73), 297 (8.68), 285 (8.98), 274 (9.17), 271 (17.07), 270 (100.00), 249 (8.68), 232 (8.98), 193 (9.46), 185 (8.98), 181 (8.68), 178 (10.34), 167 (13.37), 129 (8.49), 127 (10.73), 122 (11.90), 120 (12.00), 115 (18.83), 93 (9.46), 92 (18.93), 78 (12.59), 77 (12.49), 66 (9.85), 52 (12.68); Anal. Calcd for C_25_H_27_N_3_O_10_: C, 56.71; H, 5.14; N, 7.94%; found: C, 56.66; H, 5.14; N, 8.01%.

*5-(5-(1',2',3',4'-Tetrahydroxybutyl)-2-methylfuran-3-yl)-4-phenyl-2H-1,2,4-triazole-3(4H)-thione* (**23**). In a round bottom flask thiosemicarbazide **20** (1.823 mmoL) was refluxed with 10% aqueous sodium hydroxide solution (20 mL) for 5 h. The reaction mixture was filtered, cooled, and neutralized by gradual addition of dilute hydrochloric acid; the white precipitate of **23** was filtered off and dried. It was recrystallized from ethanol as white crystals. Yield 83%; mp 217–218 °C; Rf: 0.4 (chloroform–methanol; 5:1; *v/v*); IR (KBr) cm^−1^: 3369 (OH, NH), 1624 (C=N); ^1^H-NMR (DMSO-*d*_6_); δ: 2.30 (s, 3H, CH_3(furan)_), 3.29–3.33 (m, 1H, H-4'b), 3.45–3.47 (m, 3H, H-3', H-2', H-4'a), 4.24–4.29 (m, 2H, 4'-OH, 3'-OH, D_2_O-exchangeable), 4.45 (bs, 1H, 2'-OH, D_2_O-exchangeable), 4.55 (d, 1H, H-1', *J*_1',2'_ = 6.00 Hz), 4.91 (d, 1H, 1'-OH, *J*_1',OH_ = 6.00 Hz, D_2_O-exchangeable), 5.48 (s, 1H, CH_(furan)_), 7.32 (m, 2H, Ar-H_(c)_), 7.49 (t, 3H, Ar-H_(b, a)_, *J* = 3.00 Hz), 13.92 (s, 1H, NH, D_2_O-exchangeable); MS: *m/z* (%), 379 (5.85, M^+^+2), 378 (10.53, M^+^+1), 377 (22.81, M^+^), 360 (12.38), 359 (30.12), 298 (8.77), 287 (26.80), 286 (100.00), 285 (30.60), 284 (10.14), 237 (7.60), 211 (7.41), 199 (9.75), 183 (7.70), 170 (7.60), 162 (7.12), 155 (8.19), 144 (8.67), 138 (7.21), 131 (8.38), 128 (8.48), 127 (8.97), 124 (8.67), 118 (9.45), 116 (7.41), 104 (9.26), 95 (9.65), 92 (7.02), 91 (8.87), 77 (31.38), 66 (8.19), 61 (14.52), 60 (7.50); Anal. Calcd for C_17_H_19_N_3_O_5_S: C, 54.10; H, 5.07; N, 11.13%; found: C, 54.22; H, 5.22; N, 11.00%.

*5-(5-(1',2',3',4'-Tetraacetoxybutyl)-2-methylfuran-3-yl)-4-phenyl-2-N-acetyl-1,2,4-triazole-3-(4H)-thione* (**24**). A solution of compound **23** (1.061 mmoL) in dry pyridine (15 mL) was treated with acetic anhydride (15 mL) and the mixture was kept overnight with occasional shaking at room temperature. Then it was poured onto crushed ice, and the acetyl derivative **24** that separated out was filtered off, washed with water and dried. Yield 97%; recrystallized from ethanol as pale yellow needles; mp 168–169 °C; Rf: 0.49 (*n*-hexane–ethyl acetate; 2:1; *v/v*); IR (KBr) cm^−1^: 1747 (OAc, N-Ac), 1622 (C=N); ^1^H-NMR (CDCl_3_); δ: 1.97, 1.99, 2.01 (3s, 12H, 4OAc), 2.50 (s, 3H, CH_3(furan)_), 2.77 (s, 3H, N-Ac), 3.98–4.04 (dd, 1H, H-4'b, *J*_4'a,4'b_ = 12.00 Hz, *J*_4'b,3'_ = 6.00 Hz), 4.10–4.16 (dd, 1H, H-4'a, *J*_4'a,4'b_ = 15.00 Hz, *J*_4'a,3'_ = 3.00 Hz), 5.03–5.07 (m, 1H, H-3'), 5.39–5.46 (m, 1H, H-2'), 5.48 (s, 1H, CH_(furan)_), 5.79 (d, 1H, H-1', *J*_1',2'_ = 3.00 Hz), 7.20–7.30 (m, 2H, Ar-H_(c)_), 7.53 (t, 3H, Ar-H_(b, a)_, *J* = 3.00 Hz); MS: *m/z* (%), 589 (1.52 , M^+^+2), 588 (3.93 , M^+^+1), 587 (10.34, M^+^), 547 (9.46), 546 (26.15), 545 (79.56), 485 (6.60), 384 (20.24), 383 (57.91), 382 (5.92), 370 (4.92), 342 (12.32), 341 (28.54), 329 (4.49), 328 (15.48), 324 (11.95), 323 (13.76), 299 (8.53), 298 (6.72), 288 (7.01), 287 (21.33), 286 (100.00), 285 (9.11), 284 (5.98), 270 (4.66), 256 (6.60), 115 (10.38), 77 (8.82), 60 (4.34), 51 (2.25); Anal. Calcd for C_27_H_29_N_3_O_10_S: C, 55.19; H, 4.97; N, 7.15%; found: C, 55.11; H, 4.99; N, 7.00%.

*5-(5-(Tetrahydro-2',3'-dihydroxyfuran-1'-yl)-2-methylfuran-3-yl)-4-phenyl-2H-1,2,4-triazole-3-(4H)-thione* (**25**). A solution of compound **23** (1.823 mmoL) was heated with aqueous acetic acid (150 mL, 10%) under reflux for 5 h. After cooling the product **25** that separated out was filtered off, washed with water and dried. Yield 80%; recrystallized from ethanol as off-white needles; mp 217–218 °C; Rf: 0.4 (chloroform–methanol; 5:1; *v/v*); IR (KBr) cm^−1^: 3407–3184 (OH, NH), 1625 (C=N); ^1^H-NMR (DMSO-*d*_6_); δ: 2.32 (s, 3H, CH_3(furan)_), 3.47–3.51 (dd, 1H, H-4'b, *J*_3',4'b_ = 3.00 Hz, *J*_4'b,4'a_ = 9.00 Hz), 3.85–3.90 (dd, 2H, H-3', H-4'a, *J*_3',4'a_ = 6.00 Hz, *J*_4'b,4'a_ = 9.00 Hz), 3.97(bs, 1H, H-2'), 4.24 (d, 1H, H-1', *J*_1',2'_ = 9.00 Hz), 4.86 (d, 1H, 3'-OH, *J*_3',OH_ = 3.00 Hz, D_2_O-exchangeable), 4.92 (d, 1H, 2'-OH, *J*_2',OH_ = 6.00 Hz, D_2_O-exchangeable), 5.53 (s, 1H, CH_(furan)_), 7.32–7.35 (m, 2H, Ar-H_(c)_), 7.49 (t, 3H, Ar-H_(b, a)_, *J* = 3.00 Hz), 13.96 (bs, 1H, NH, D_2_O-exchangeable); MS: *m/z* (%), 360 (23.69, M^+^+1), 359 (20.21, M^+^), 357 (20.21), 341 (20.21, M^+^-H_2_O), 326 (20.21, M^+^-SH), 321 (26.48), 316 (24.74), 314 (24.74), 305 (24.39), 289 (29.97), 255 (20.91, M^+^-C_4_H_8_O_3_), 225 (26.48), 212 (25.44), 203 (25.78), 167 (25.78), 153 (27.53), 149 (32.06), 141 (27.53), 139 (24.74), 129 (25.78), 118 (24.74), 112 (25.44), 111 (25.44), 97 (30.31), 95 (29.27), 94 (50.52), 93 (27.53), 90 (25.78), 83 (43.21), 81 (33.80), 74 (29.97), 73 (30.31), 71 (56.10), 70 (33.80), 69 (49.48), 67 (24.74), 61 (24.74), 60 (45.99), 57 (100.00), 56 (42.16), 55 (76.66), 54 (24.39), 52 (25.78); Anal. Calcd for C_17_H_17_N_3_O_4_S: C, 56.81; H, 4.77; N, 11.69%; found: C, 56.77; H, 4.77; N, 11.74%.

*4-(4,5-Dihydro-4-phenyl-5-thioxo-1H-1,2,4-triazol-3-yl)-5-methylfuran-2-carbaldehyde* (**26**). A solution of compound **23** (2.122 mmol) in distilled water (20 mL) was treated dropwise with a solution of sodium metaperiodate (6.366 mmol) in distilled water (20 mL) with continuous stirring for 5 h, the formyl derivative **26** that separated out was filtered off, washed with water, and dried. Yield 48%; recrystallized from ethanol as pale yellow needles; mp 237–238 °C; Rf: 0.77 (chloroform–methanol; 20:1; *v/v*); IR (KBr) cm^−1^: 3354 (NH), 1681 (CHO), 1600 (C=N); ^1^H-NMR (DMSO-*d*_6_); δ: 2.46 (s, 3H, CH_3(furan)_ with DMSO), 6.65 (d, 1H, CH_(furan)_), 7.39 (t, 2H, Ar-H_(c)_, *J* = 3.00 Hz), 7.50–7.57 (m, 3H, Ar-H_(b, a)_), 9.32 (s, 1H, CHO), 14.17 (s, 1H, NH, D_2_O exchangeable); MS: *m/z* (%), 287 (6.12, M^+^+2), 286 (19.64, M^+^+1), 285 (100.00, M^+^), 284 (33.49), 256 (19.21), 228 (17.85), 212 (6.13), 169 (9.29), 150 (7.92), 149 (16.37), 135 (6.34), 134 (13.27), 118 (6.39), 109 (7.29), 106 (6.53), 93 (6.00), 91 (11.22), 80 (68.01), 79 (9.20), 78 (13.83), 77 (61.00), 76 (7.14), 69 (6.12), 66 (7.25), 65 (13.54), 64 (43.58), 63 (10.89), 55 (5.63), 53 (6.78), 52 (14.86), 51 (47.93), 50 (13.88); Anal. Calcd for C_14_H_11_N_3_O_2_S: C, 58.93; H, 3.89; N, 14.73%; found: C, 58.90; H, 4.00; N, 14.88%.

### 3.3. Antioxidant and Anticancer Screening

#### 3.3.1. Materials

Mammalian cell lines: MCF-7 cells (human breast cancer cell line were obtained from VACSERA Tissue Culture Unit (Cairo, Egypt). Chemicals used: Dimethyl sulfoxide (DMSO), crystal violet and trypan blue dye were purchased from Sigma (St. Louis, MO, USA). Fetal bovine serum, Dulbecco’s Modefied Eagle’s Medium (DMEM), RPMI-1640, HEPES buffer solution, L-glutamine, gentamycin and 0.25% trypsin-EDAT were purchased from Lonza (St. Louis, MO, USA). Crystal violet (1%) was made from 0.5% (w/v) crystal violet and 50% methanol, then made up to volume with dd H_2_O and filtered through a Whatman No. 1 filter paper.

#### 3.3.2. Cell Line Propagation

The cells were propagated in (DMEM) supplemented with 10% heat-inactivated fetal bovine serum, 1% l-glutamine, HEPES buffer and 50 µg/mL gentamycin. All cells were mentained at 37 °C in humidified atmosphere with 5% CO_2_ and were subcultured two times a week. Cell toxicity was monitored by determining the effect of the examined compound on cell morphology and cell viability.

#### 3.3.3. Cytotoxicity Evaluation Using Viability Assay

For the cytotoxicity assays, cells were seeded in 96-well plate at a cell concentration of 1 × 10^4^ cell per well in 100 μL of growth medium. Fresh medium containing different concentrations of the test sample was added after 24 h of seeding. The microtiter plates were incubated at 37 °C in a humidified incubator with 5% CO_2_ for a period of 48 h. Three wells were used for each concentration of the tested sample. Control cells were incubated without test sample and with or without DMSO. After incubation of the cells for 24 h at 37 °C, various concentrations of the sample (50.000, 25.000, 12.500, 6.250, 3.125 and 1.560 μg) were added each separately. The incubation was continued for 48 h and viable cells yield was determined colorimetrically using 3,4,5-dimethylthiazol-2-yl-2,5-diphenyltetrazolium bromide (MTTB). The water insoluble tetrazolium salt is converted to purple formazan by the mitochondrial dehydrogenase of viable cells. After the end of incubation period, media were aspirated and crystal violet solution (1%) was added to each well for at least 30 min. The stain was removed and plates were rinsed using tap water until all excess stain is removed. Glacial acetic acid (30%) was then added to all wells and mixed thoroughly, then the absorbance of the plates were measured after gently shaken on Microplate Reader (Tecan, Inc., city, country), at 490 nm. All results were corrected for background absorbance detected in wells without added stain. Treated sample was compared with the cell control in the absence of the tested compound. All experiments were carried out in the triplicate. The cell cytotoxic effect of the tested compound was calculated [27,28].

## 4. Conclusions

Some new aromatic *C*-nucleosides have been prepared from carbohydrate precursors. Their physical and chemical properties were studied, and some of the compounds showed potential antioxidant activities. One of these compounds has been screened for its antitumor activity.
